# Double-Resonant Nanostructured Gold Surface for Multiplexed Detection

**DOI:** 10.1021/acsami.1c23438

**Published:** 2022-01-28

**Authors:** Antonio Minopoli, Emanuela Scardapane, Bartolomeo Della Ventura, Julian A. Tanner, Andreas Offenhäusser, Dirk Mayer, Raffaele Velotta

**Affiliations:** † Department of Physics “E. Pancini”, University Federico II, Via Cintia 26, 80126 Naples, Italy; ‡ Institute of Biological Information Processing (IBI-3), Bioelectronics, Forschungszentrum Jülich, 52425 Jülich, Germany; § School of Biomedical Sciences, University of Hong Kong, Hong Kong, China

**Keywords:** plasmon-enhanced fluorescence, multiplexing, photochemical immobilization technique, apta-immunosensor, gold nanoparticles, nanostructured surface, block copolymer micelle nanolithography

## Abstract

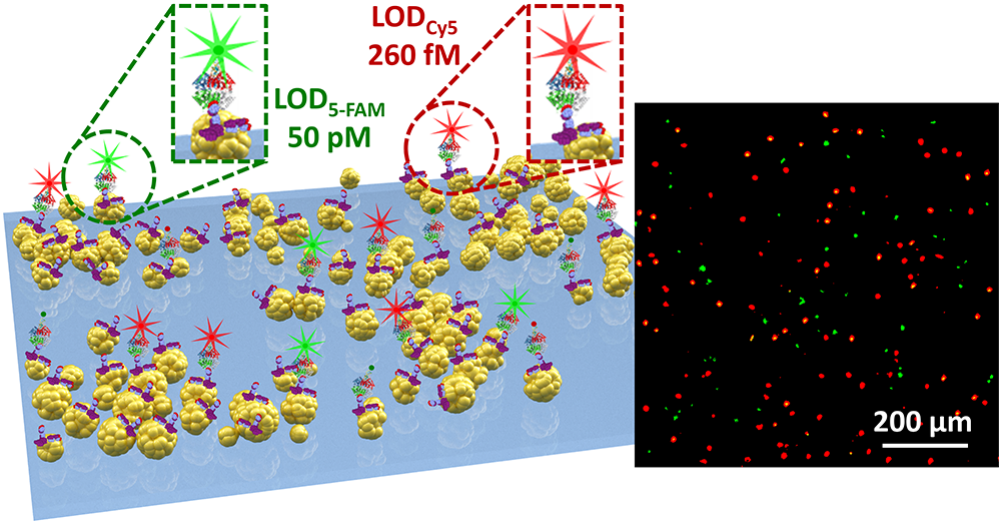

A novel double-resonant plasmonic substrate for fluorescence amplification in a chip-based apta-immunoassay is herein reported. The amplification mechanism relies on plasmon-enhanced fluorescence (PEF) effect. The substrate consists of an assembly of plasmon-coupled and plasmon-uncoupled gold nanoparticles (AuNPs) immobilized onto a glass slide. Plasmon-coupled AuNPs are hexagonally arranged along branch patterns whose resonance lies in the red band (∼675 nm). Plasmon-uncoupled AuNPs are sprinkled onto the substrate, and they exhibit a narrow resonance at 524 nm. Numerical simulations of the plasmonic response of the substrate through the finite-difference time-domain (FDTD) method reveal the presence of electromagnetic hot spots mainly confined in the interparticle junctions. In order to realize a PEF-based device for potential multiplexing applications, the plasmon resonances are coupled with the emission peak of 5-carboxyfluorescein (5-FAM) fluorophore and with the excitation/emission peaks of cyanine 5 (Cy5). The substrate is implemented in a malaria apta-immunoassay to detect *Plasmodium falciparum* lactate dehydrogenase (*Pf*LDH) in human whole blood. Antibodies against *Plasmodium* biomarkers constitute the capture layer, whereas fluorescently labeled aptamers recognizing *Pf*LDH are adopted as the top layer. The fluorescence emitted by 5-FAM and Cy5 fluorophores are linearly correlated (logarithm scale) to the *Pf*LDH concentration over five decades. The limits of detection are 50 pM (1.6 ng/mL) with the 5-FAM probe and 260 fM (8.6 pg./mL) with the Cy5 probe. No sample preconcentration and complex pretreatments are required. Average fluorescence amplifications of 160 and 4500 are measured in the 5-FAM and Cy5 channel, respectively. These results are reasonably consistent with those worked out by FDTD simulations. The implementation of the proposed approach in multiwell-plate-based bioassays would lead to either signal redundancy (two dyes for a single analyte) or to a simultaneous detection of two analytes by different dyes, the latter being a key step toward high-throughput analysis.

## 1 Introduction

In the last few years, plasmonic nanostructures are routinely used to amplify the signal in plasmon-enhanced fluorescence (PEF),^1^ surface plasmon-coupled emission (SPCE),^2^ surface-enhanced Raman scattering (SERS),^3^ and surface-enhanced infrared absorption (SEIRA) applications.^4^ In particular, the fluorescence amplification is highly desirable in biosensing applications to remarkably lower the detection limits, notably in complex biological systems exhibiting significant interfering background (e.g., autofluorescence, crosstalk, competing signals).^5^ Even though the physics underlying the PEF phenomenon is still to be fully comprehended,^6^ the fluorescence enhancement (FE) can be ascribable to the optical coupling between the nanostructure and the fluorescent molecules.^1^ The particular enhancement mechanism (i.e., excitation rate enhancement, emission rate enhancement, dual-mechanism enhancement) is determined by the (i) fluorophore-nanostructure spectral overlap and (ii) fluorophore-nanostructure separation distance.^7,8^ In general, if the plasmonic resonance overlaps the fluorophore excitation, the local modification of the electromagnetic field induced by the nanostructure results into an increase of the fluorophore excitation rate due to the Förster resonance energy transfer (FRET) effect. In the case of overlap with the fluorophore emission, the nanostructure alters the radiative and non-radiative decay rates through the Purcell effect and, hence, the fluorescence lifetime and quantum yield. Actually, the nanostructure works as a resonant cavity that amplifies the fluorescence emission on-resonance while quenches it off-resonance. This is due to the modification of the local density of states (LDOS) induced by the cavity.^9^ In real cases, both FRET and Purcell effects usually take place leading to a strong quenching at a few nanometer fluorophore-nanostructure separation distance, a large FE in the range of 5–30 nm (with an optimum at ∼10 nm), and a weak coupling at longer distances.

Rough metal surfaces constitute one of the simplest tools to realize a fluorescence enhancer for biosensing applications since they generally offer high densities of randomly distributed electromagnetic hot spots on macroscopic areas. Additionally, employing a sensing area with no discontinuities provides the substrate with a multitude of anchoring sites for biomolecules to make the bioreceptor-substrate binding easier. Nevertheless, their moderate FEs limit their applicability to bioassays whose interest concerns concentrations larger than tens of picomolar.^10^

Thus, a wide variety of nanostructured platforms have been recently explored to devise high-performance fluorescence enhancers apt to realize ultrasensitive bioassays such as nanoassemblies,^11,12^ nanocages,^13,14^ and nanopatterns.^15−17^ Particularly, Soret colloids realized through thermo-driven self-assembly^18^ represent an intriguing approach to achieve not only remarkable FEs (up to thousands-fold),^19,20^ thanks to the collective and coherent coupling between localized surface plasmons and surface plasmon polaritons,^19,21^ but also highly directional and *p*-polarized emission enhancements (>97%) when implemented on SPCE platforms.^19,22^ Even larger FEs (up to 10^6^-fold)^23^ were measured by using particular designs sustaining strong interplasmon coupling such as nanocavities^17,24^ and nanoantennas.^25,26^ However, such architectures require a nanoscale positioning of the fluorescent molecules at electromagnetic hot spots to make their adoption in real biosensing applications unpractical.^27^

Two-dimensional (2D) patterns of metal nanoparticles represent a smart solution to simultaneously augment both the FEs via interplasmon coupling and the hot spot density. Their optical properties crucially depend on lattice parameters and on the size, shape, material, and immediate environment of nanoparticles.^28^ In fact, modifications in the index of refraction of the immediate environment entail a variation of the optical response so that nanostructured surfaces can be effectively used for bulk refraction index sensing and molecular sensing.^29−31^ As it concerns the material, silver is generally preferred to gold in fluorescence-based assays because of the lower quenching entailed by silver at nanoscale distance (<5 nm). However, the significant chemical reactivity of silver makes it prone to oxidation and dissolution, which is detrimental for some applications in real matrices.^32^ On the other hand, gold exhibits high biocompatibility and inertness.^33,34^ Innovative strategies have been recently explored for dequenching the fluorophore emission, such as adopting photonic crystal and graphene oxide as substrates.^35,36^ In this regard, photonic crystal-coupled emission (PCCE) platforms were effectively employed to reduce the quenching, thereby improving the fluorescence of about 100-fold and 1500-fold with silver nanoparticles (AgNPs) and gold nanoparticles (AuNPs), respectively.^19,37^

Patterns of AuNPs are valid candidates as a signal enhancer since they combine tunable plasmonic features with simple fabrication.^38−40^ In a first approximation, their optical behavior depends on the ratio *D*/*d* between the particle diameter (*D*) and the center-to-center distance (*d*). When a such a value exceeds 2/5,^41^ a plasmonic coupled mode, whose resonance is generally red-shifted in accordance with the hybridization model,^42,43^ can arise as a result of the near-field interaction among the localized surface plasmons (LSPs). On the contrary, when *D*/*d* < 2/5, the optical behavior of the nanoparticle pattern can be deduced from a system of optically decoupled LSPs.^41^

Additionally, multi-resonant plasmonic modes can be activated by properly tailoring the pattern architecture so that these structures are suitable for multiplexed bioanalytical assays. Multiplexing-based assays are highly appealing in diagnostics since they benefit from lower detection time, sample volume, and costs despite generally suffering from low sensitivity and specificity, also requiring complex microfluidic systems, sample pretreatments, and purification steps.^44,45^

As it concerns the nanoparticle patterning, plenty of methods were recently developed to fabricate periodic arrays of AuNPs on large scale areas.^38,46,47^ Self-assembly is a smart technique to efficiently arrange a large number of nanoparticles onto macroscopic surfaces. Additionally, arbitrary patterns can be conveniently obtained by first self-assembling the nanoparticles onto lithography-fabricated templates. Such a nanoimprinting approach successfully fabricated nanoparticle patterns with single particle resolution.^48^ A promising alternative is represented by colloid lithography. In this case, the nanoparticles are first packed to form a mono-layer (generally at air/solvent interface) and then transferred onto the substrate (e.g., by etching, dip-coating, or lift-off).^49,50^

When fabrication affordability and scalability as well as optical tunability are required, block copolymer micelle nanolithography (BCMN) stands out over other methods thanks to its capability to easily produce large-scale periodic arrays of AuNPs whose lattice parameters can be modified by simply choosing the appropriate diblock copolymers.^51^ In recent studies, we successfully realized two plasmonic substrates consisting of hexagonally arranged^52^ (utilizing BCMN) and randomly positioned^53^ AuNPs (electrostatic immobilization) apt to be implemented in a PEF-based apta-immunoassay for detecting malaria biomarker *Plasmodium falciparum* lactate dehydrogenase (*Pf*LDH) in human whole blood down to femtomolar and picomolar levels, respectively, without any sample preconcentration and pretreatment.

The quest for multiplexed detection, while preserving high quality performances, spurred us to devise a double-resonant plasmonic nanostructure suitable for simultaneously detecting two different analytes in the matrix of interest. Thus, we tailored BCMN in such a way to fabricate branch patterns made of plasmon-coupled hexagonally arranged AuNPs, which gives rise to a coupled mode whose resonance lies in the far-red region, and sprinkled plasmon-uncoupled AuNPs that exhibit an LSP resonance (LSPR) at 524 nm. As a case study, we implemented the proposed plasmonic nanostructure in a PEF-based malaria apta-immunosensor for detecting *Pf*LDH in spiked whole blood. The *Pf*LDH is a biomarker secreted by the *Plasmodium falciparum* parasite, the most common and lethal among the malaria parasites (90% of malaria-related mortality worldwide).^54^

The PEF-based apta-immunoassay herein described combines the intrigued optical properties of a double-resonant plasmonic nanostructure with a robust antibody-functionalization strategy, the so-called photochemical immobilization technique (PIT).^55^The latter was proven to be capable to covalently bind antibodies (Abs) on gold surfaces in an orientated way so that the one fragment antigen-binding (Fab) site can explore the immediate environment.^55,56^ While Abs were preferred as a capture bioreceptor layer since the simple and effective functionalization carried out via PIT, fluorescently labeled aptamers (Apts*) were employed as the top bioreceptor layer in the sandwich configuration to (i) significantly increase the specificity, (ii) enable optimal separation distance between fluorophore and nanostructure (approximately 10 nm), and (iii) accomplish a versatile and affordable fluorescent labeling of the analytes of interest. It is worth mentioning that our approach allowed us to not be overly concerned about dequenching strategies since fluorophores were inherently positioned beyond the FRET region.

## 2 Results and Discussion

### 2.1 Characterization of the Substrate

#### 2.1.1 Morphology

**Figure 1:**
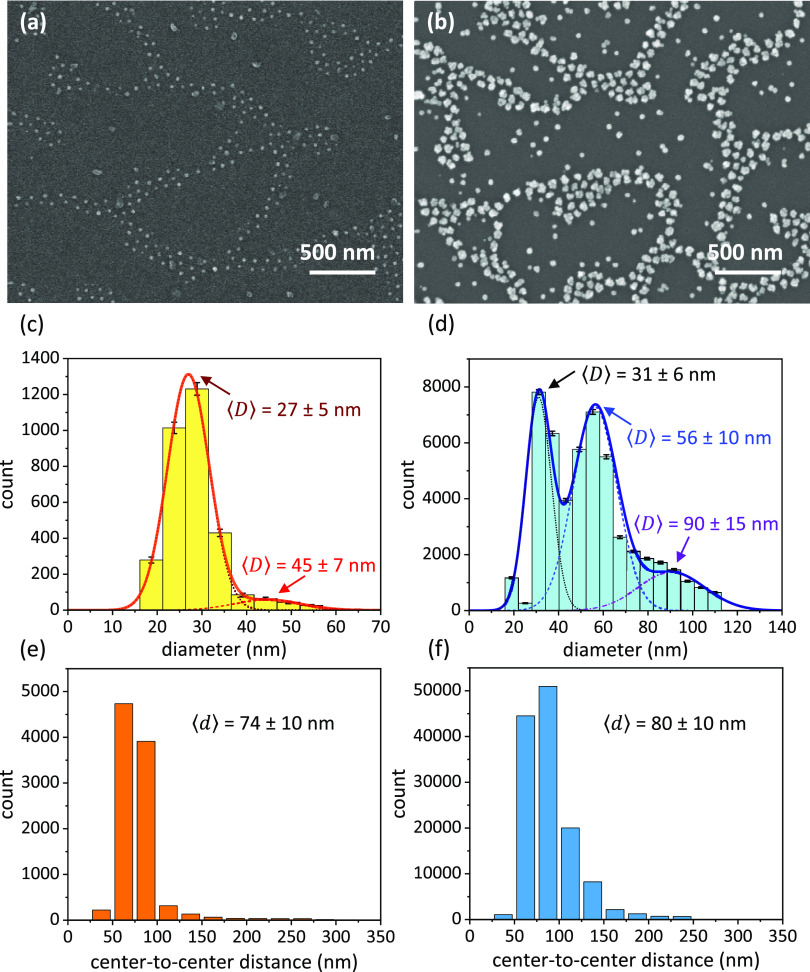
SEM images of the substrate (a) before and (b) after the nanoparticle growth. Histograms of the nanoparticle diameter (c) before and (d) after the nanoparticle growth. The solid orange and blue lines are the fits obtained by considering the histograms as the sum of (c) two and (d) three Gaussian distributed populations, respectively. Histograms of the center-to-center distance (e) before and (f) after the nanoparticle growth.

The morphological characterization of the substrate was accomplished by scanning electron microscopy (SEM) (details are reported in Section S1). Figure 1a depicts a SEM image at high magnification of the nanostructured pattern. Aiming to activate the plasmonic coupled modes of the AuNPs arranged along the branches, the particle growth was carried out to increase the *D*/*d* value (Figure 1b). A higher number of isolated AuNPs appears as a by-product of the growth process. Instead of representing a detriment, such isolated AuNPs trigger a localized resonance mode in addition to the coupled mode. The histogram of the nanoparticle size before the growth process includes two Gaussian distributed populations: patterned AuNPs whose distribution is peaked at 27 nm with a standard deviation of 5 nm and isolated larger gold by-products randomly distributed onto the substrate whose size is 45 ± 7 nm (Figure 1c). The histogram after the nanoparticle growth includes three populations: isolated AuNPs whose diameter is 31 ± 6 nm, larger AuNPs of 56 ± 10 nm diameter arranged along the branches, and isolated gold by-products of 90 ± 15 nm size (Figure 1d). The center-to-center distance distribution of patterned AuNPs did not significantly change after the nanoparticle enlargement meaning that the growth process does not alter the patter architecture (Figure 1e,f). An average center-to-center distance of 80 nm was large enough to sustain a plasmonic coupled mode for patterned AuNPs of approximately 50–60 nm diameter.

#### 2.1.2 Optical Response

**Figure 2:**
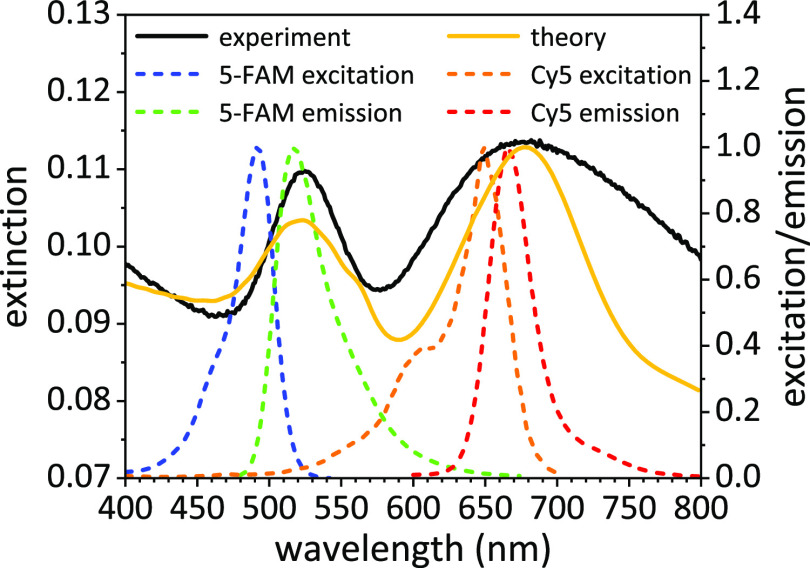
Experimental (solid black line) and theoretical (solid gold line) extinction spectrum of the substrate. Plasmon-fluorophore spectral overlap with 5-FAM (emission coupling, dashed green line) and Cy5 (dual-mechanism coupling, dashed orange and red lines) dyes.

The experimental extinction spectrum of the substrate exhibits two resonances at (i) 524 and (ii) 675 nm (solid black line in Figure 2). (i) Isolated AuNPs give rise to the localized mode at 524 nm, as expected of AuNPs of 30 nm diameter in air,^57,58^ whereas (ii) patterned AuNPs entail a coupled mode at 675 nm.^57^ The finite-difference time-domain (FDTD) method was adopted to solve Maxwell’s equations in order to retrieve the theoretical response of the substrate when stimulated by an external electromagnetic perturbation. The simulated curve (solid gold line in Figure 2) consistently reproduces that experimentally observed (solid black line in Figure 2) (technical details on FDTD simulations are reported in Section S2). For comparison, we also worked out the extinction spectra of the nanostructure without combination of AuNPs with different sizes (see Section S3).

Once the substrate was optically characterized, the fluorophore should be generally chosen so that its excitation/emission peaks overlap with the plasmon extinction.^1,8^ Given the large variety of organic fluorophores apt to this aim, the spectral overlap may be virtually accomplished at any wavelengths in the visible range. In this regard, we selected 5-carboxyfluorescein (5-FAM) and cyanine 5 (Cy5) dyes whose excitation/emission spectra are reported in Figure 2. While the narrow resonance at 524 nm restricts the spectral overlap to the only radiative coupling with 5-FAM dye (emission peak at 520 nm, dashed green line), the broad resonance band at higher wavelengths leads to a dual-mechanism coupling with Cy5 dye (excitation/emission 650 nm/665 nm, dashed orange and red lines).

It is worth mentioning that the choice of 5-FAM dye was not optimal since its excitation peak lay off-resonance (490 nm). In such a way, we did not fully exploit the amplification potential of the plasmonic resonance at 524 nm. Indeed, a fluorophore whose excitation peak was in the green band may experience a higher FE as compared to the 5-FAM dye.

### 2.2 Apta-Immunoassay Sensing Performance

#### 2.2.1 Gold Surface Biofunctionalization by PIT

The substrates were PIT-functionalized with pan malaria Abs (anti-*P*LDH). First, the anti-*P*LDH concentration was varied over a large range to optimize the surface covering (Figure S4a). Both the plasmonic resonances red-shifted as the anti-*P*LDH concentration increased up to 50 μg/mL, above which the substrate is unable to house more Abs. Thus, an anti-*P*LDH concentration of 50 μg/mL was used throughout all the experiments yielding a plasmon red-shift of approximately 4–5 nm due to the dielectric protein layer surrounding the AuNPs (Figure S4b). The efficient surface filling is also demonstrated by the lack of significant optical change in the extinction spectrum after the blocking step (dashed red line in Figure S4b). Since the steric hindrance of a PIT-immobilized Ab is approximately150 nm^2^,^59^ the average number of Abs per nanoparticle of 60 nm (30 nm) diameter was ∼75 (∼20).

#### 2.2.2 Detection Scheme

**Figure 3:**
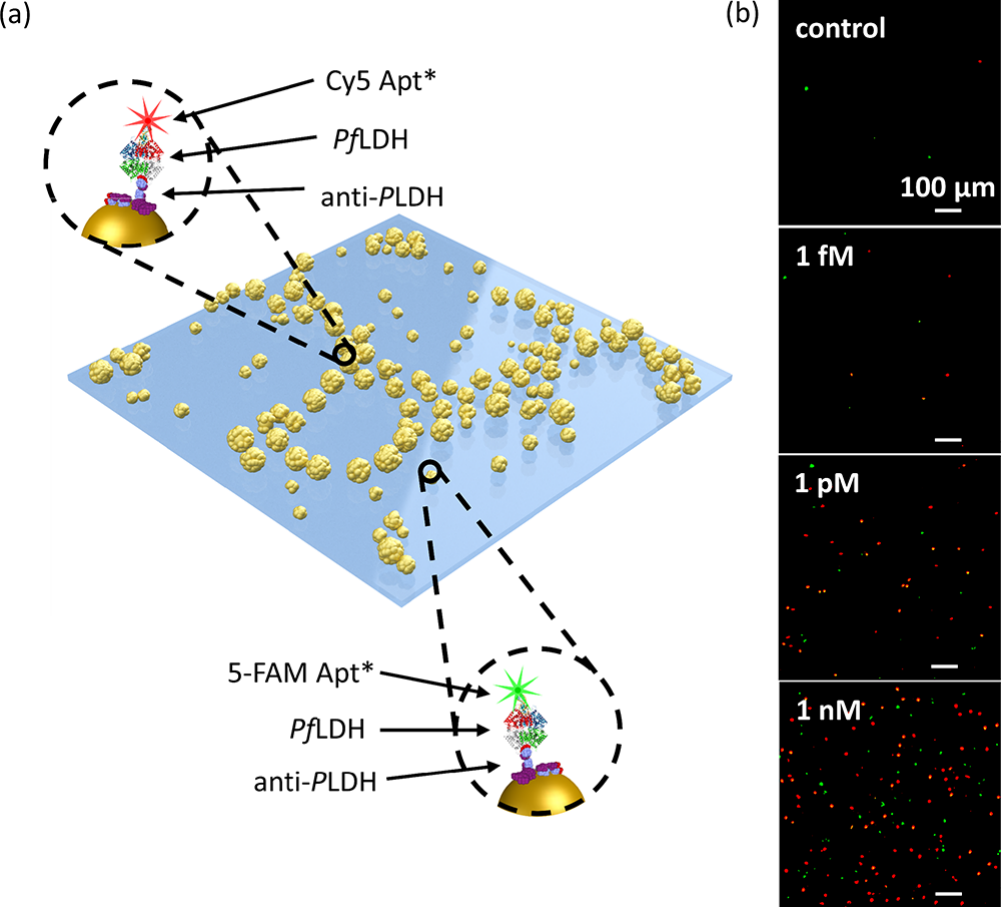
(a) Sketch of the pattern architecture consisting of branch hexagonally arranged and sprinkled AuNPs. The insets show the Ab-*Pf*LDH-Apt* sandwich schemes in the case of 5-FAM and Cy5 labels. (b) Example of fluorescence images recorded at different *Pf*LDH concentrations spiked in human whole blood.

This work was conceived as a case study for evaluating the performance of the proposed substrate as a fluorescence enhancer in a PEF-based biosensor in multiplexed measurements for simultaneously detecting two different analytes. The multiplexed detection was obtained by adopting the sandwich configuration Ab-analyte-Apt* shown in Figure 3a. The capture bioreceptor layer was realized by well-oriented Abs with one Fab exposed to the surrounding environment thanks to the PIT functionalization, thereby significantly increasing the effectiveness of the analyte binding. Two kinds of labeled aptamers (with green and red probes) were used as a recognition bioreceptor layer to explore the double-resonance of the branch pattern.

Figure 3b shows some representative fluorescence pictures at different analyte concentrations. Notably, the bright spot number is clearly distinct from the control (i.e., uninfected human whole blood) down to the picomolar level for both the fluorophores (full details about acquisition and processing of fluorescence pictures are reported in Section S5). Note that the fluorescence of each bright spot is likely to arise from one single fluorophore as explained in detail in Section S6.

#### 2.2.3 Calibration Curves

**Figure 4:**
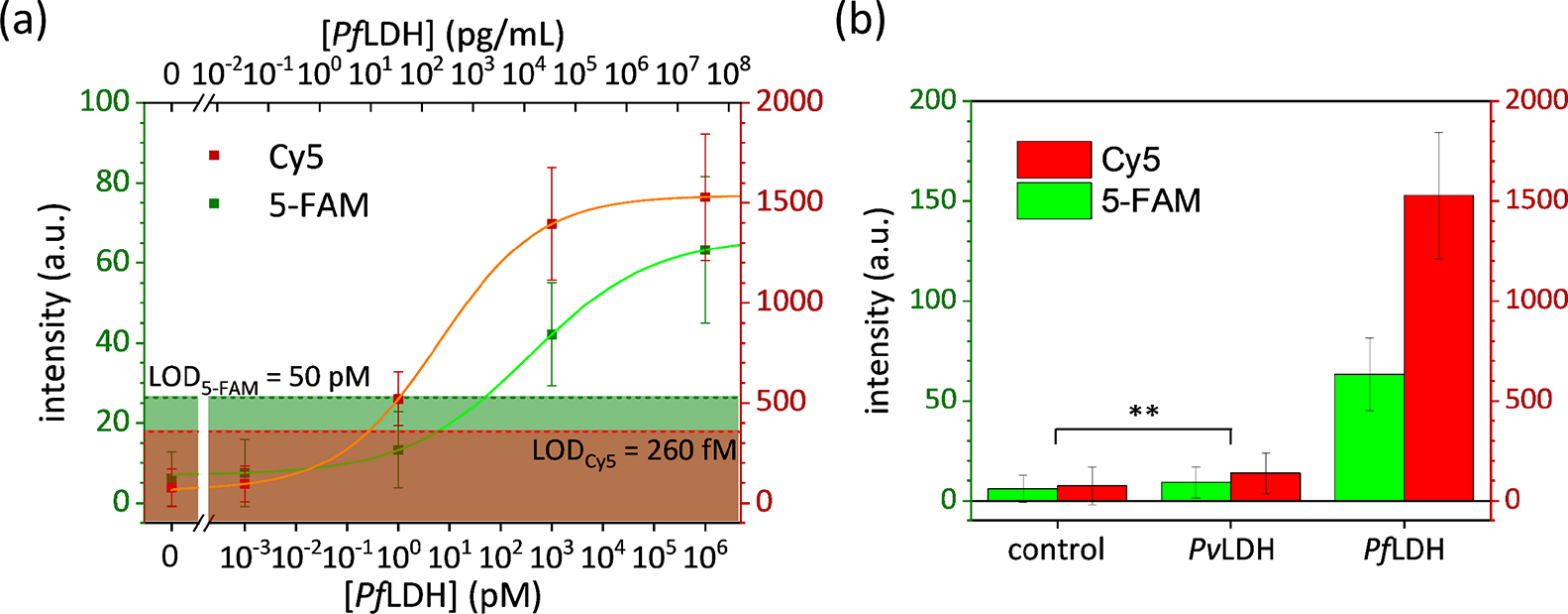
(a) Fluorescence intensity as a function of *Pf*LDH concentration spiked in human whole blood (calibration curve). The best fit curves (solid green and orange lines) are the four-parameter Hill eq 1. The shaded regions represent the 3σ noise level measured in uncontaminated whole blood. (b) Specificity of the apta-immunoassay against the *Pv*LDH (**p-value < 0.001). The data are averaged on ten measurements and are reported as mean value ± standard deviation.

The dependence of the fluorescence intensity *F* on the analyte concentration is shown in Figure 4a (details about fluorescence analysis are reported in Section S5). The data are fitted by the four-parameter Hill equation^60^
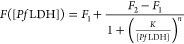
1where *F*_1_ and *F*_2_ are the minimum and maximum value of the fluorescence intensity, respectively, *K* is the concentration at which the fluorescence is equal to half of its maximum value, and *n* is the so-called Hill’s coefficient.^61^Table 1 reports the best-fit parameter values, the linear range (LR), and the limit of detection (LOD), estimated as 3σ above the control value, for both the fluorescent dyes used in this work.

**Table 1:** Best-Fit Parameter Values, LR, and LOD Obtained by Fitting the Experimental Data of Figure 4a with Eq 1

parameter	5-FAM	Cy5
*F*_1_	7 ± 1 arb. units	6 ± 2 arb. units
*F*_2_	66 ± 3 arb. units	154 ± 4 arb. units
*K*	(0.36 ± 0.15) × 10^3^ pM	6 ± 1.7 pM
*n*	0.36 ± 0.05	0.44 ± 0.06
χ^2^	1.2	1.8
LR	10 pM–1 μM	100 fM–1 nM
LOD	50 pM (1.6 ng/mL)	260 fM (8.6 pg/mL)

For comparison, we listed in Table 2 some recently reported platform-based biosensors for multiplexed measurements. It is remarkable to note that in many applications, the multiplexing was realized by performing parallel measurements on one platform containing different chips, each of them devoted to one analyte of interest. However, although such an approach constitutes a smart strategy to achieve multiplexed analysis without sophisticate multi-response chips, it may require complex microfluidic systems or an increase of specimen amount and costs.^44,45^ On the contrary, chips exhibiting multi-responsivity only need one specimen to carry out multiplexed analysis thereby reducing materials and costs.^62,63^

**Table 2:** Overview on Recently Reported Platform-Based Biosensors for Multiplexed Analysis^a^

platform	method	matrix	analytes	LR (pM)	LOD (pM)	remarks	ref
AuNPs-modified screen-printed carbon electrode	EIS	protease solution	*Listeria monocytogenes*	10–10^7^ CFU mL^–1^	9 CFU mL^–1^	multiplexed measurements were performed on a microarray of four electrodes functionalizing each of them accordingly to the analyte of interest	(68)
			*Staphylococcus aureus*	10–10^7^ CFU mL^–1^	3 CFU mL^–1^		
nanostructured conductive hydrogel electrodes on PET film	amperometry	human serum	triglycerides	0.1–6 × 10^9^	7×10^7^	multiplexed measurements were performed on a prescreen-printed electrode unit containing three screen-printed carbon paste working electrodes. Each electrode was functionalized against the analyte of interest	(67)
			lactate	0.08–5 × 10^9^	6 × 10^7^		
			glucose	1–25 × 10^9^	2 × 10^8^		
glass	interferometry	human serum	anti-thyroglobulin IgG	6–400 IU mL^–1^	6 IU mL^–1^	multiplexed measurements were performed on a multi-spot sensor chip functionalizing each spot properly	(66)
			anti-thyroid peroxidase IgG	1.7–860 IU mL^–1^	1.7 IU mL^–1^		
Au triangular nanoprisms on MPTMS functionalized	LSPR	human plasma	microRNA-10b	10^–4^–10^4^	5.9 × 10^–5^	multiplexed measurements were carried out in a 96-well plate. Each well was used for detecting one analyte	(44)
			microRNA-96	10^–4^–10^4^	5.9 × 10^–5^		
			microRNA-145	10^–4^–10^4^	2.12 × 10^–4^		
			microRNA-143	10^–4^–10^4^	1.86 × 10^–4^		
			microRNA-490-5p	10^–4^–10^4^	1.83 × 10^–4^		
AgNP film on silicon wafer	Raman spectroscopy	PBS	α-fetoprotein	5.9–4300^b^	0.1*c*	LR and LOD were estimated in 100× diluted samples. Thus, the results should be worsened by two orders of magnitude when referred to real samples	(65)
			glypican-3	5.9–4300^b^	0.1^c^		
Au core–SERS label–Ag shell–Au shell on silicon wafer	Raman spectroscopy	PBS	microcystin-LR	10–10^4^	1.5	multiplexed measurements were performed on one chip functionalized with two different probes consisting of aptamer-driven core–satellite assemblies	(63)
			microcystin-RR	10–10^4^	1.3		
Au-coated grating	fluorescence	PBS (1 mg/mL BSA)	anti-mouse IgG-AF790	0.01–5	6 × 10^–3^	multiplexed measurements may be carried out on one chip by using microfluidic channels	(64)
branch pattern of AuNPs on glass coverslip	fluorescence	human whole blood	*Pf*LDH-5-FAM	10–10^6^	50	the effectiveness of multiplexed analysis was demonstrated by evaluating the double-response of the chip with two fluorescent tags labeling one kind of analyte	this work
			*Pf*LDH-Cy5	0.1–10^3^	0.26		

a EIS: electrochemical impedance spectroscopy, PET: polyethylene terephthalate, MPTMS: (3-mercaptopropyl)-trimethoxysilane, PBS: phosphate-buffered saline, BSA: bovine serum albumin, AF790: Alexa Fluor 790.

b Data not given in the reference; the value was estimated by the calibration curve.

c LOD not given in the reference; the value reported in the table corresponds to the lowest measured analyte concentration.

#### 2.2.4 Specificity

The specificity of the proposed apta-immunosensor was tested against the *Plasmodium vivax* lactate dehydrogenase (*Pv*LDH) (90% residue identity with the *Pf*LDH).^69^ To this aim, the desired amount of *Pv*LDH (1 μM referred to undiluted blood) was spiked into the diluted specimens (human whole blood from healthy donors 1:100 diluted in 1 mL of 25 mM Tris buffer). Figure 4b illustrates the fluorescence intensity measured in human whole blood with no analyte (control), competitive analyte (*Pv*LDH), and analyte of interest (*Pf*LDH). Although the bottom bioreceptor layer, consisting of pan malaria anti-*P*LDH, can capture any *Plasmodium* malaria marker, the high selectivity of malaria aptamers warranted a negligible cross-reaction with *Pv*LDH.^70^

### 2.3 Fluorescence Enhancement

#### 2.3.1 Electromagnetic Simulations

We investigated the electromagnetic response of the substrate when interacting with a plane wave radiation **E_0_**. The ratio between the intensity of the electric field induced by the nanostructure, **E**, and the intensity of the incident radiation is defined as the gain factor *G*(71)
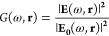
2where ω is the frequency of the impinging perturbation and **r** is the position vector.

**Figure 5:**
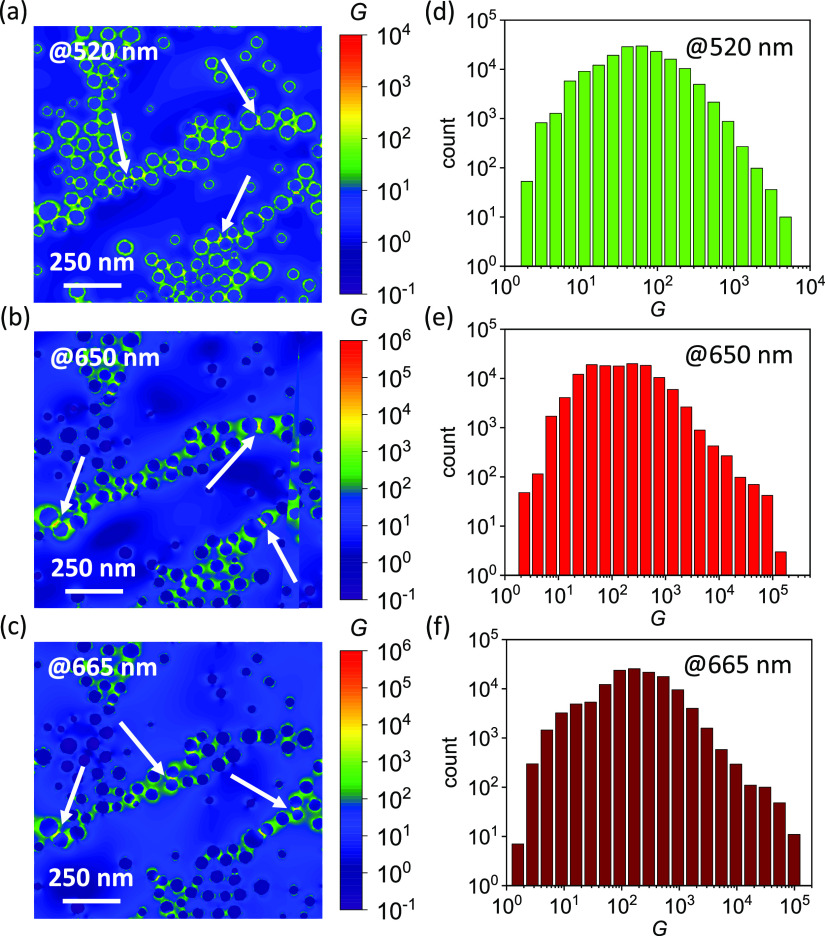
Simulated electric field intensity of the substrate worked out at wavelength (a) 520, (b) 650, and (c) 665 nm. The white arrows point to some plasmonic hot spots of the nanostructure. Histograms of the *G* value distribution evaluated in annulus-shaped regions around nanoparticles (5 nm thickness, 5 nm offset from particle surface) at wavelengths (d) 520, (e) 650, and (f) 665 nm.

We modeled the nanostructure according to the architecture morphology provided by SEM images. In addition, we implemented a surface roughness onto each nanoparticle to approach the observed nanoparticle shape (see Section S2B for details). Since the size of the fluorophores we used is ∼1 nm^3^,^72^ we discretized the substrate over a mesh with 1 nm spatial resolution so that a dye can fit in a unit cell. Figure 5a–c shows the intensity distributions of the electric field at the emission peak of 5-FAM (520 nm) and at the excitation/emission peaks of Cy5 (650/665 nm), whereas those worked out off-resonance (490 and 575 nm) are depicted in Figure S7.

Regions surrounding nanoparticles exhibit higher *G* values as compared to the free space as a result of the strong confinement of the electric field: These regions are generally called hot spots.^71^

At 520 nm wavelength, the main contribution to the electric field arose from the dipolar modes of nanoparticles. In this case, the electric field was mainly enhanced at the nanoparticle edges (along the polarization direction) or confined at the nanoparticle dimer junctions (Figure 5a). However, such a plasmonic mode (i.e., not coupled mode) yielded a relatively low amplification (*G* values do not exceed few thousand). On the contrary, much higher *G* values (up to hundreds of thousands) were observed at 650 and 665 nm wavelengths as a result of the strong interplasmon coupling among nearby AuNPs (Figure 5b,c). A possible way to capture the physics underlying the coupled modes relies on the so-called plasmon hybridization method,^42,73^ according to which the modes resulting from a single nanoparticle mix (hybridize) with those from the nearby nanoparticles giving rise to bonding and antibonding plasmonic modes.^43^

Since we aimed at using this platform for sensing applications where fluorophores are placed at a distance of 5–10 nm from the surfaces of the nanoparticles, we focused our analysis on these annulus-shaped regions around each nanoparticle (details are reported in Section S8). Figure 5d,e shows the *G* value distributions in these regions at wavelengths of 520, 650, and 665 nm, respectively. The histograms reveal that only a tiny fraction of fluorophores would experience a relatively high *G* value meaning that most of them were positioned in places whose local amplification may not be strong enough to yield a measurable fluorescence.

Additionally, we worked out the electromagnetic response of the substrate with perfectly spherical nanoparticles (see Section S2A for details). In this case, much lower *G* values were attained revealing that the surface roughness was a crucial feature to augment the local field (Figure S9).^74^ For comparison, we also investigated the electromagnetic response of different nanoparticle shapes and materials in isolated and hexagonally arranged configurations.^75^ For a futuristic scope, it is worth mentioning that up to 100-fold amplification could be potentially achieved with sharp silver nanoparticles as compared to smooth gold nanoparticles (see Section S10).

#### 2.3.2 Comparison between Theoretical and Measured Fluorescence Enhancement

The FE factor is defined as^71^
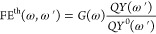
3where *QY* is the fluorophore quantum yield in PEF conditions, *QY*^0^ is the fluorophore quantum yield in free-space, and ω and ω′ are the excitation and emission frequency, respectively. We aimed at comparing the FE^th^ values at excitation/emission wavelengths of the fluorophores with those experimentally measured (later described). Since *QY*^0^ is an inherent parameter of the fluorophore, the ratio *QY*/*QY*^0^ is bound above due to the constraint *QY*≤ 1. Considering *QY*_5FAM_^0^ = 0.83 at 520 nm wavelength for 5-FAM dye^76^ and *QY*_Cy5_^0^ = 0.27 at 665 nm wavelength for Cy5 dye,^77^ the ratio *QY*/*QY*^0^ cannot exceed 1.2 for the 5-FAM dye and 3.7 for Cy5. Thus, the *G* value distributions shown in Figure S7c and Figure 5e can be immediately converted into FE̅^th^ distributions at 490/520 and 650/665 nm excitation/emission, respectively.

**Figure 6:**
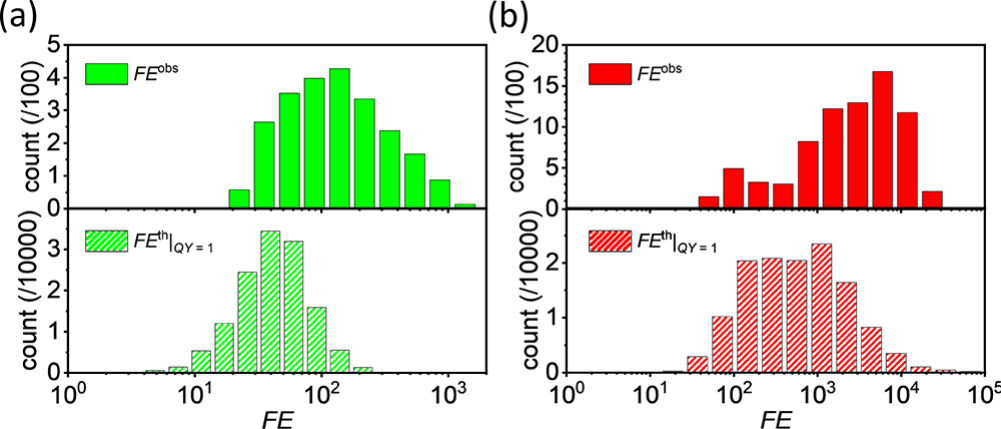
Experimental (top subpanels) and theoretical (bottom subpanels) FE distributions for (a) 5-FAM and (b) Cy5 dyes. Theoretical distributions are worked out at (a) 490/520 and (b) 650/665 nm excitation/emission wavelengths in the case *QY* = 1. The experimental histograms were obtained over an area of 1.66 × 1.40 mm^2^, whereas the theoretical histograms were worked out over an area of 1.25 × 1.25 μm^2^.

As it concerns the measured FE factor, it can be estimated as^78^
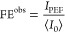
4where *I*_PEF_ is the fluorescence intensity provided by fluorophores in the presence of the nanostructure and ⟨*I*_0_⟩ is the mean fluorescence signal of fluorophores under non-PEF conditions (details are reported in Section S11). Figure 6a,b shows the FE^obs^ distributions for 5-FAM and Cy5 dyes, respectively (top subpanels). Thus, we can compare such distributions with FE̅^th^ distributions worked out in the case *QY* = 1 (bottom subpanels).

A slight discrepancy emerges between FE^obs^ and FE̅^th^ distributions in both the fluorophore channels probably ascribable to an underestimated nanoparticle roughness in the simulation modeling. Note that such a discrepancy would rise if perfectly spherical nanoparticles were considered (see Section S9) rather than rough nanoparticles, thus corroborating the crucial role played by the nanoparticle roughness in enhancing the fluorescence.

An estimation of the average FE factor, ⟨FE^obs^⟩, can be retrieved by^78^
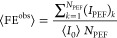
5where *N*_PEF_ is the number of bright spots. Equation 4 yields ⟨FE_5FAM_^obs^⟩ = 160 for 5-FAM dye and ⟨FE_Cy5_^obs^⟩ = 4500 for the Cy5 dye. It is worth mentioning that the ratio between ⟨FE_Cy5_^obs^⟩ and ⟨FE_5FAM_^obs^⟩ of approximately 28 is consistent with the ratio between the slopes of the corresponding correlation curves (fluorescence vs spot area) (see Section S6).

## 3 Conclusions

In this paper, we presented a novel double-resonant plasmonic substrate for potential multiplexed and high-throughput analysis in PEF-based biosensing. The substrate consists of an assembly of hexagonally arranged and sprinkled AuNPs, which gives rise to a double resonance at 524 and 675 nm wavelengths. The former was coupled with the emission peak of the 5-FAM dye and the latter with both the excitation and emission peaks of the Cy5 dye. Numerical simulations demonstrated that the pattern architecture endowed the substrate with a large amount of intense electromagnetic hot spots in which fluorophores can be housed.

The substrate was implemented in a malaria apta-immunoassay for detecting *Pf*LDH in human whole blood. We adopted Abs as capture bioreceptor layer and Apts* as the top fluorescently labeled layer. Substrate functionalization was realized through PIT, which warranted that Abs covalently tethered to nanoparticle surfaces in well-oriented configuration. In addition, the adoption of Apts* rather than fluorescently labeled Abs drastically reduced the cost per assay while improving the specificity. The *Pf*LDH was simultaneously detected by both the fluorophores as proof of concept for multiplexed analysis. Additionally, the simultaneous detection of two fluorescent probes can provide high signal redundancy and an extension of the detection range.

We achieved competitive LODs of 260 fM with the Cy5 dye and 50 pM with the 5-FAM dye. No complex sample pretreatments are required making such a device suitable for point of care tests. The measured average FE values of 160 (with 5-FAM dye) and 4500 (with Cy5 dye) are consistent with those simulated by considering branch patterns of rough AuNPs. It is worth mentioning that the LOD may be improved up to 100-fold in a transparent matrix such as human serum or plasma since 1:100 dilution required for whole blood would be not necessary.

Finally, the BCMN fabrication technique is extremely versatile allowing one to easily tune the plasmonic response in the visible range. As futuristic scope, multi-resonant devices may be also conceived by properly tailoring the pattern architecture or combining different metal nanoparticles in such a way to active additional plasmonic modes. Moreover, sharp nanoparticles may further augment the electromagnetic field by at least one order of magnitude as compared to smooth nanoparticles as simulations revealed. The potential biosensing applications of the proposed approach are far-reaching, for not only multi-analyte detection but also biomarker panel identification with double signal redundancy.

## 4 Experimental Section

### 4.1 Chemicals and Materials

A comprehensive list is reported in Section S12.

### 4.2 Substrate Fabrication and Characterization

#### 4.2.1 Fabrication of Branch Patterns of AuNPs

**Figure 7:**
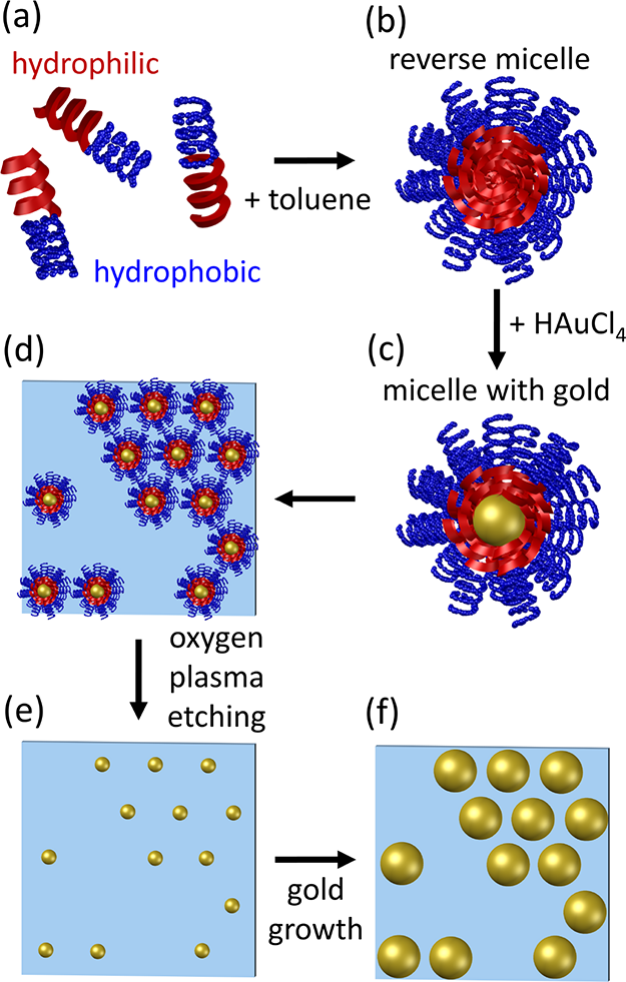
Fabrication scheme of the branch pattern of AuNPs by BCMN. (a) Dispersion of amphiphilic diblock copolymers in nonpolar solvent. (b) Self-assembly of reverse micelles. (c) Formation of Au seeds inside the hydrophilic core. (d) Laying of PS-AuNPs on the substrate. (e) Sticking of the AuNPs onto the glass slide after the copolymer etching. (f) Enlargement of the nanoparticle size.

BCMN was adopted to fabricate branch patterns of hexagonally arranged AuNPs over a large area.^51^ The procedure included four steps sequentially shown in Figure 7.

An amount of 24.3 mg of diblock copolymer P3807-S2VP (Figure 7a) was dispersed into 15 mL of toluene. The solution was kept under vigorous stirring for 72 h to obtain a homogeneous monodisperse solution of reverse micelles (Figure 7b). An amount of 13.1 mg of HAuCl_4_·3H_2_O was loaded into the solution under vigorous stirring for 72 h to allow the inception of Au seeds (inside the micelle core) covered by polystyrene shells (PS-AuNPs) (Figure 7c). The solution appears yellowish. Then, possible copolymer aggregates were removed by filtering the solution. Diblock copolymers and gold(III) chloride trihydrate were handled in a glovebox under inert gas (argon) and controlled conditions (O_2_ < 1 ppm, H_2_O < 0.1 ppm).

Before the PS-AuNP deposition onto the substrate, glass coverslips (10 × 8 mm^2^) were sonicated for 5 min in acetone, 2-propanol, and ethanol sequentially to remove dust and impurities. Afterward, the cleaned substrates were dipped in a nonpolar solvent (toluene) to enable the sticking of hydrophobic polystyrene shells. Then, the substrates were vertically dipped into the solution of PS-AuNPs by means of a dip-coater to warrant an extremely fine positioning and speed control. The dipping speed was set to 0.8 mm/s. Such a speed is low enough to warrant PS-AuNP laying onto the substrate while preventing the maximum close-packing (Figure 7d). Finally, an oxygen plasma treatment (0.8 mbar, 200 W, 30 min) was used to etch the copolymers so to leave the AuNPs immobilized onto the substrates (Figure 7e).

Afterward, the substrates were incubated with 2 mL of gold growth solution (CTAB 190 mM, HAuCl_4_·3H_2_0 42 mM, AgNO_3_ 8 mM, ascorbic acid 100 mM) for 2 h enabling the increase of AuNP size while holding center-to-center distances (Figure 7f).^38^

#### 4.2.2 Analysis of Scanning Electron Micrograph

Full details are reported in Section S1.

#### 4.2.3 Numerical Simulations

Technical details on FDTD simulations are reported in Section S2.

### 4.3 Fluorescence Apta-Immunoassay

#### 4.3.1 Surface Biofunctionalization and Blocking

Gold surface functionalization with pan malaria anti-*P*LDH was realized through the well-established PIT.^55,56^ Full details about the PIT are reported in Section S13.

The aqueous solution containing anti-*P*LDH (50 μg/mL concentration, 1 mL volume) was UV irradiated for 30 s (6 W at 254 nm) and conveyed onto the substrate. The latter was integrated in a microfluidic system consisting of an interacting cell housing the substrate, a 2 mL syringe, and Tygon tubes with a diameter of 1 mm (for both the input and output channel) designed for biological samples (Figure S13). The volume of the solution in contact with the substrate was ∼30 μL, whereas the total volume flowing into the circuit was approximately 200 μL. The syringe was used to repeatedly draw 250 μL from the fresh solution containing the irradiated Abs (4 draws separated by a time interval of 3 min). Then, ultrapure water was copiously flowed into the circuit to remove the unbound Abs from the substrate.

The blocking step was carried out by flowing into the circuit an aqueous solution of 50 μg/mL BSA (4 draws of 250 μL separated by a time interval of 1 min). Afterward, ultrapure water was copiously flowed into the circuit to remove unbound BSA molecules. Finally, the substrates were stored in PBS solution at room temperature (see Section S15 for details about PBS preparation).

#### 4.3.2 Ab-Analyte-Apt* Stacking

Blood specimens were drawn from the healthy donors via monovette tubes. Ethylenediaminetetraacetic acid (EDTA) was added to prevent blood coagulation. Whole blood was diluted 1:100 in 25 mM Tris buffer to reduce the turbidity of the specimen (see Section S15 for details about Tris buffer preparation). The analyte was spiked into 1 mL of the diluted specimen to achieve a *Pf*LDH concentration in the range 1 fM–1 μM (referred to undiluted whole blood). Control experiments were performed in uncontaminated specimens (diluted whole blood).

The functionalized substrates stored in the buffer solution (ready-to-use) were slightly rinsed by ultrapure water and then incubated with *Pf*LDH-spiked specimens (diluted whole blood) by gently shaking the sample for 2 h to improve the *Pf*LDH capture efficiency by immobilized Abs. Afterward, the substrates were abundantly rinsed by ultrapure water and Tris buffer to remove blood residues and unbound *Pf*LDH molecules.

5-FAM- and Cy5-labeled malaria Apt* were added in the ratio 1:1 into 1 mL of PBS (10 mM) to achieve a final Apt* concentration of 0.1 μM. Thus, the substrates were transferred into the solution by gently shaking the bowl for 2 h in dark conditions, thereby realizing the Ab-*Pf*LDH-Apt* sandwich scheme shown in Figure 3a. Then, the substrates were copiously rinsed by ultrapure water and PBS to remove unbound Apts*.

#### 4.3.3 Fluorescence Picture Processing and Analysis

Full details are reported in Section S5.

## Supporting Information Available

Analysis of scanning electron micrographs (Section S1); coding of numerical simulations (Section S2); optical response of homogeneously sized gold nanoparticles (Section S3); substrate biofunctionalization (Section S4); fluorescence picture acquisition, processing, and analysis (Section S5); analysis of the bright spots (Section S6); off-resonance simulations (Section S7); analysis of the electric field (Section S8); electromagnetic response of the substrate with perfectly spherical nanoparticles (Section S9); electromagnetic response of non-spherical nanoparticles (Section S10); fluorescence intensity in non-PEF conditions (Section S11); chemicals and materials (Section S12); photochemical immobilization technique (Section S13); microfluidic system (Section S14); preparation of buffer solutions (Section S15) (PDF)
