# Supplementary material for: Double-Resonant Nanostructured Gold Surface for Multiplexed Detection
Source: ACS Appl Mater Interfaces. 2022 Jan 28;14(5):6417–27. doi: 10.1021/acsami.1c23438 (PMC8832399; doi:10.1021/acsami.1c23438)
Supplement: Supplementary file 1 — am1c23438_si_001.pdf [file am1c23438_si_001.pdf]

Supporting Information

Double-resonant nanostructured gold surface for multiplexed detection

Antonio Minopoli,[1,2] Emanuela Scardapane,[1] Bartolomeo Della Ventura,[1] Julian A. Tanner,[3] Andreas Offenhäusser,[2] Dirk Mayer,[2,*] and Raffaele Velotta[1,*]

[1]Department of Physics “E. Pancini”, University Federico II, Via Cintia 26, 80126 Naples, Italy.

[2]Institute of Biological Information Processing (IBI-3), Bioelectronics, Forschungszentrum Jülich, 52425 Jülich, Germany.

[3]School of Biomedical Sciences, University of Hong Kong, Hong Kong SAR, China.

*Corresponding authors: rvelotta@unina.it, dirk.mayer@fz-juelich.de

Table of contents

Section S1. Analysis of scanning electron micrographs.

Section S2. Coding of numerical simulations.

Section S3. Optical response of homogeneously sized gold nanoparticles.

Section S4. Substrate biofunctionalization.

Section S5. Fluorescence picture acquisition, processing and analysis.

Section S6. Analysis of the bright spots.

Section S7. Off-resonance simulations.

Section S8. Analysis of the electric field.

Section S9. Electromagnetic response of the substrate with perfectly spherical nanoparticles.

Section S10. Electromagnetic response of non-spherical nanoparticles.

Section S11. Fluorescence intensity in non-PEF conditions.

Section S12. Chemicals and materials.

Section S13. Photochemical immobilization technique.

Section S14. Microfluidic system.

Section S15. Preparation of buffer solutions.

References.

Section S1. Analysis of scanning electron micrographs.

SEM images were acquired by Zeiss LEO 1550VP field emission scanning electron microscope with a nominal resolution of 1 nm at 20 kV acceleration voltage by collecting secondary electrons with an In-Lens detector. The morphology of the substrate was analysed processing the micrographs by ImageJ software. Firstly, objects were isolated from the background by thresholding the raw image (Figures S1a and S1b), then adjacent nanoparticles were segmented by watershedding (Figure S1c). "Analyze Particles" tool implemented in ImageJ was run to retrieve information about the object perimeter p, area S, shape descriptors, and centroid coordinates. Figure S1d shows an example of processed micrograph in which the nanoparticles are decomposed in outline and inner area. Afterwards, nanoparticle diameter was estimated as $D = 2\sqrt{S/\pi}$, whereas the centre-to-centre distance distribution was carried out by calculating the distance of each centroid from its nearest neighbours.

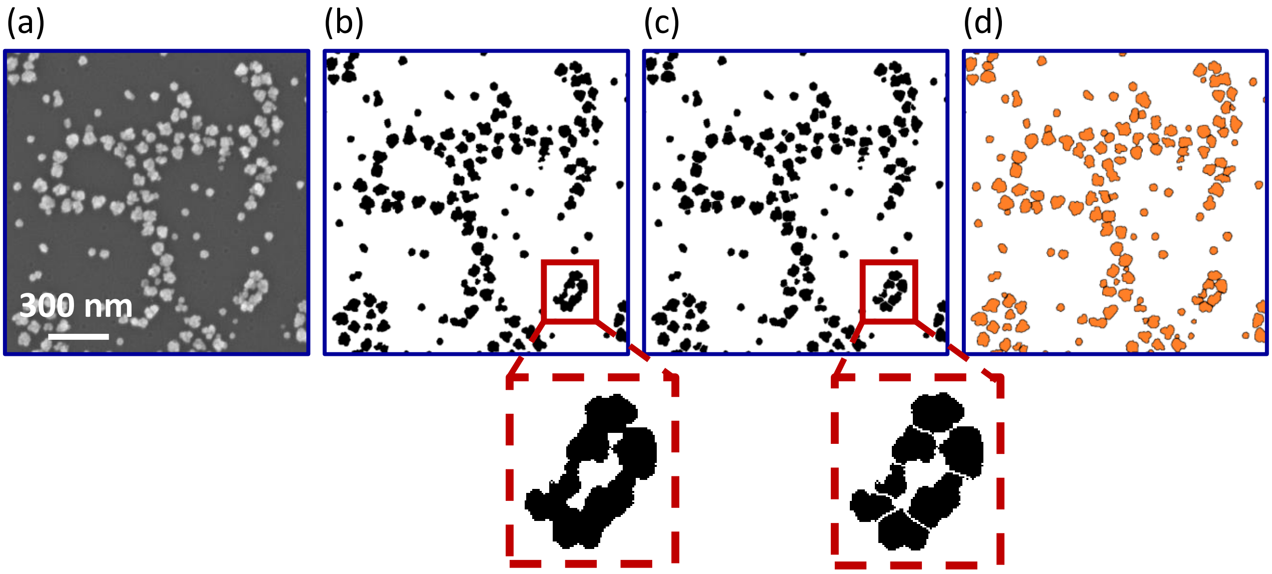

Figure S1. (a) Example of raw SEM micrograph at high magnification. Corresponding (b) thresholded and (c) segmented image. The insets show a magnification of a nanoparticle cluster highlighting the capability to effectively segment adjacent objects by the watershedding algorithm. (d) Processed image in which the nanoparticles are decomposed in outlines (black line) and inner region (orange filling).

Section S2. Coding of numerical simulations.

The optical response of the nanostructured substrate was simulated by using the finite-difference time-domain (FDTD) method implemented in “FDTD solutions” tool of Lumerical software. The Maxwell’s equations are numerically solved in the time domain within a Mie problem-like framework by discretizing the space over a mesh and evaluating the evolution of the electric and magnetic fields in each cell.

Section S2.A. Branch pattern of spherical AuNPs.

An illustration of the simulation workspace is shown in Figure S2a. The optical behaviour of the substrate was explored through linearly polarized waves (400-800 nm wavelength, 0-180° polarization angle with angular step of 15°) propagating along *z* direction. A photodetector placed in the *x*-*y* plane was dedicated to measure the electric field over time (1.25×1.25 μm^2 size) (not shown in Figure S2a), whereas a second photodetector positioned on the opposite side of the workspace was set to collect the transmitted photons to work out the extinction spectrum of the nanostructure (400-800 nm wavelength, 1 nm spectral resolution). The nanostructure was realized by positioning homogeneous gold spheres[1] onto a layer of silicon dioxide[2] (200 nm thickness) according to the real morphology provided by SEM images. Nanoparticle centroids and diameters were retrieved by raw SEM images as described in Section S1. Then, homogeneous gold spheres were shaped in the Lumerical environment using “addsphere” script command. The refractive index of surrounding environment was set equal to 1.

Periodic boundary conditions (BCs) were set along *x* and *y* directions to extend the simulation over an infinite array. Bloch BCs were used for simulations with non-zero polarization angles in order to compensate the phase shift arising when an electromagnetic disturbance with a non-zero angle should be reinjected at the opposite workspace site. Perfect matched layer BCs along *z* direction (steep angle profile, 12 layers) warranted the complete absorption of the wave backscattered through the light source and that traveling beyond the photodetector.[3] The spatial resolution of the mesh was set equal to 1 nm to warrant high accuracy of the results while holding the simulation time within few hours.

Section S2.B. Branch pattern of rough AuNPs.

Technical parameters of simulations were set as in the Section S2.A. In addition, we implemented a degree of roughness onto the surface of spheres. Firstly, homogenous gold spheres were placed onto a layer of silicon dioxide[2] (200 nm thickness) according to the real

morphology. The diameters were reduced of 20 nm as compared to the real size (see Section S1). Secondly, gold hemispheres of radius 10 nm were randomly arranged onto the spherical surfaces allowing interpenetration. More precisely, 20 (40) (80) (120) (160) (200) gold hemispheres were placed onto spheres whose real diameter lied in the range 10-20 nm (20-30 nm) (30-40 nm) (40-60 nm) (60-80 nm) (> 80 nm). The size of the resulting rough spheres as well as their degree of roughness was comparable with the real one observed by electron microscopy (Figure S2b).

Section S2.C. Hexagonal cell of non-spherical nanoparticles.

The optical response of non-spherical nanoparticles was explored through a *x*-polarized wave (300-800 nm wavelength) propagating along *z* direction. A photodetector placed in the *x*-*y* plane was dedicated to measure the electric field over time (160×138 nm^2 size), whereas a second photodetector positioned on the opposite side of the workspace was set to collect the transmitted photons to work out the extinction spectrum of the nanostructure (300-800 nm wavelength, 1 nm spectral resolution).

Nanoparticles of different shape (sphere, disk, ring, cube, rhombus, octahedron) and material (copper[1], silver[1], gold[1]) were arranged in hexagonal cells (80 nm lattice constant) (Figure S2c). Non-spherical nanoparticles were added into Lumerical framework using default 3D models. Also, the response of isolated nanoparticles was studied for comparison. The refractive index of surrounding environment was set equal to 1. The geometrical parameters and lattice constant of the investigated nanostructures are listed in Table S1.

Symmetric/anti-symmetric BCs set along *x* and *y* direction extended the plasmonic response over an infinite 2D array while reduced the simulation time by a factor 8 without worsening the accuracy of the results. Perfect matched layer BCs set in *z* (steep angle profile, 12 layers) assured perfect absorption of the electromagnetic waves backscattered through the plane containing the light source and incident upon the opposite side of the workspace.[3] The spatial resolution of the mesh was set equal to 1 nm to warrant high accuracy of the results while holding the simulation time within few hours.

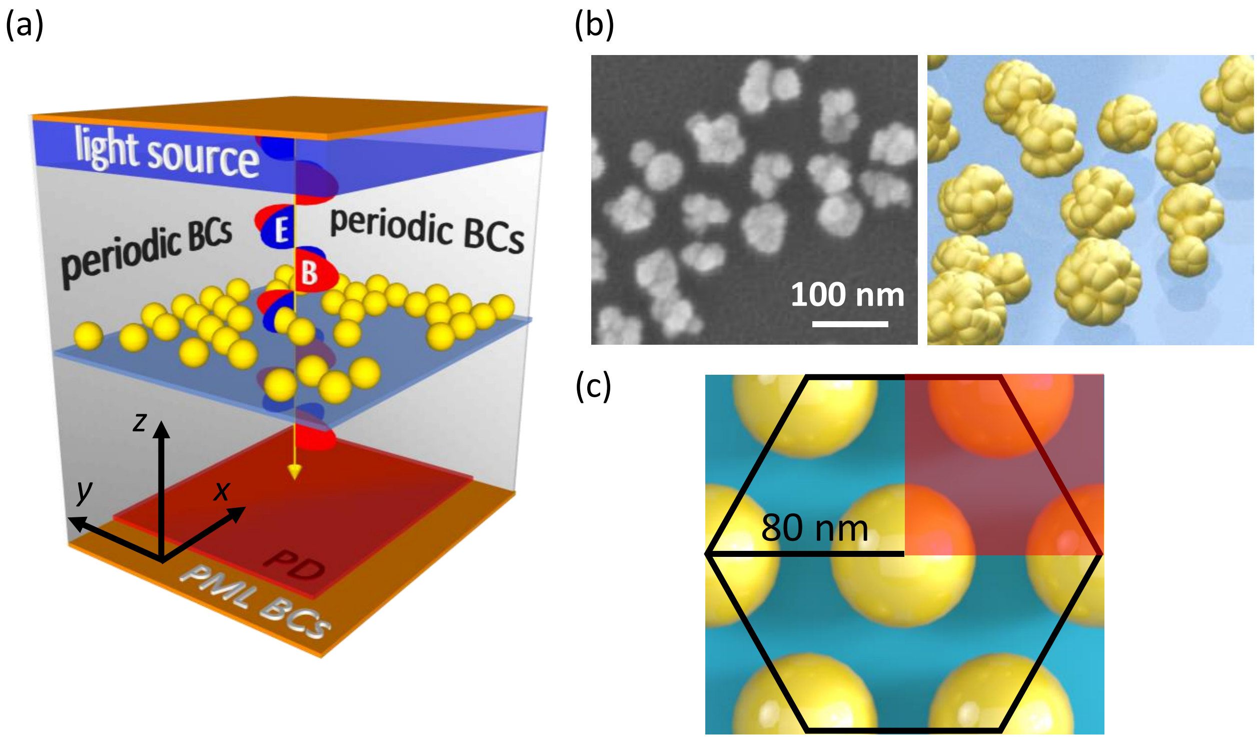

Figure S2. (a) Sketch of the simulation workspace consisting of light source, plasmonic nanostructure, dielectric substrate, photodetector (PD), and appropriate boundary conditions (BCs). (b) Comparison between the real morphology of the substrate observed by SEM (left panel) and a representative model employed in FDTD simulations (right panel). (c) Example of Au spheres arranged in a hexagonal cell. The shaded red rectangle represents the region in which the Maxwell's equations are numerically solved. Then, the solutions are symmetrically/anti-symmetrically extended to the whole hexagonal cell.

Table S1. Geometrical and lattice parameters of the investigated nanostructures. Dimensions of hexagonally arranged nanoparticles correspond to those of isolated nanoparticles and hence they are omitted to improve the readability.

**Isolated nanoparticles**			**Hexagonally arranged nanoparticles**		
**Au**	**Ag**	**Cu**	**Au**	**Ag**	**Cu**
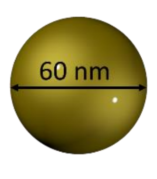	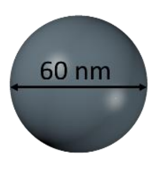	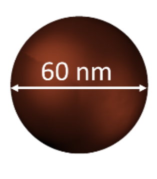	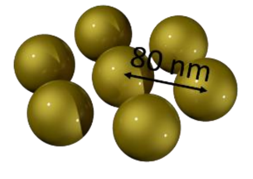	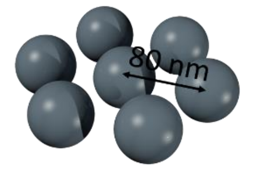	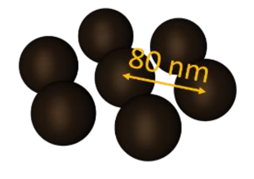
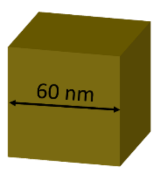	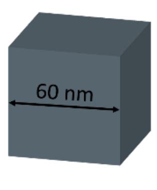	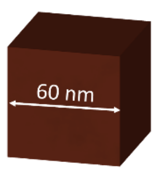	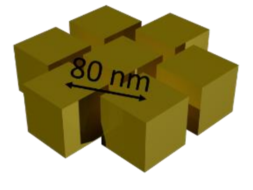	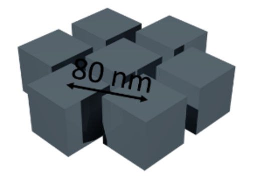	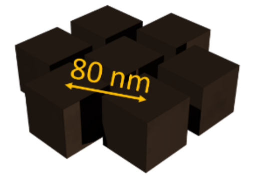
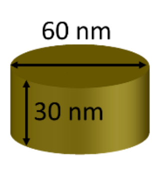	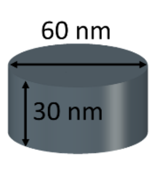	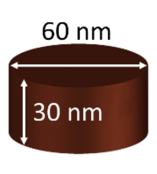	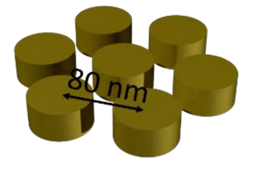	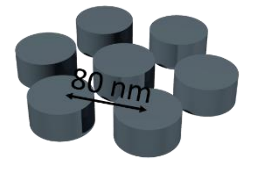	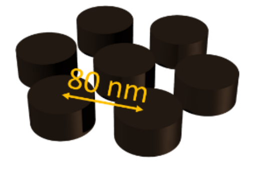
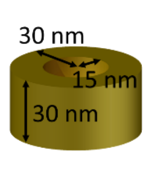	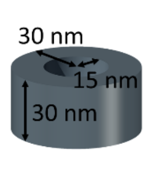	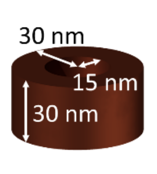	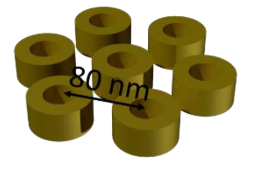	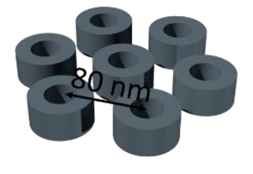	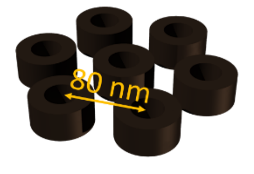
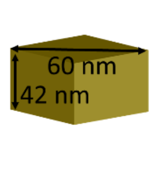	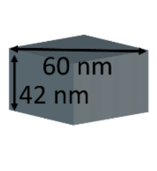	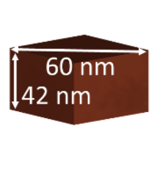	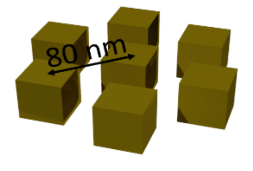	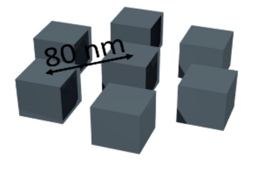	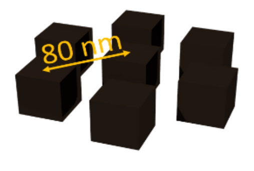
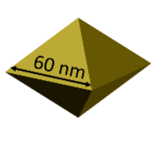	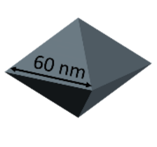	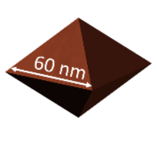	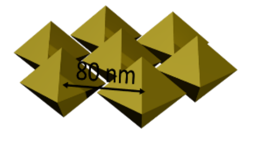	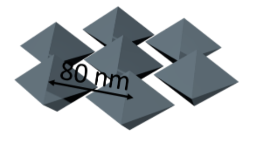	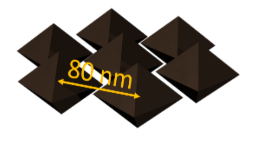

Section S3. Optical response of homogeneously sized gold nanoparticles.

Numerical simulations worked out with homogeneously sized AuNPs confirmed that the measured double resonance of the real substrate is due to not only the branch architecture, but also the combination of heterogeneously sized AuNPs. Actually, the optical response of a pattern made of 30 nm diameter AuNPs conveys a broad peak in the extinction spectrum at 540 nm wavelength. This plasmonic mode may contain both the response of isolated nanoparticles (at ∼520 nm) and that of plasmon-coupled nanoparticles (Figures S3a, S3b and S3c). On the contrary, the pattern made of 60 nm diameter AuNPs exhibits two resonances at 540 nm and 675 nm. Plasmon-uncoupled AuNPs contribute to the first plasmonic mode, whereas plasmon-coupled AuNPs produce the second mode (Figures S3d, S3e and S3f). Note that we coded the script for placing homogeneously sized 60 nm diameter AuNPs in such a way to avoid particle interpenetration.

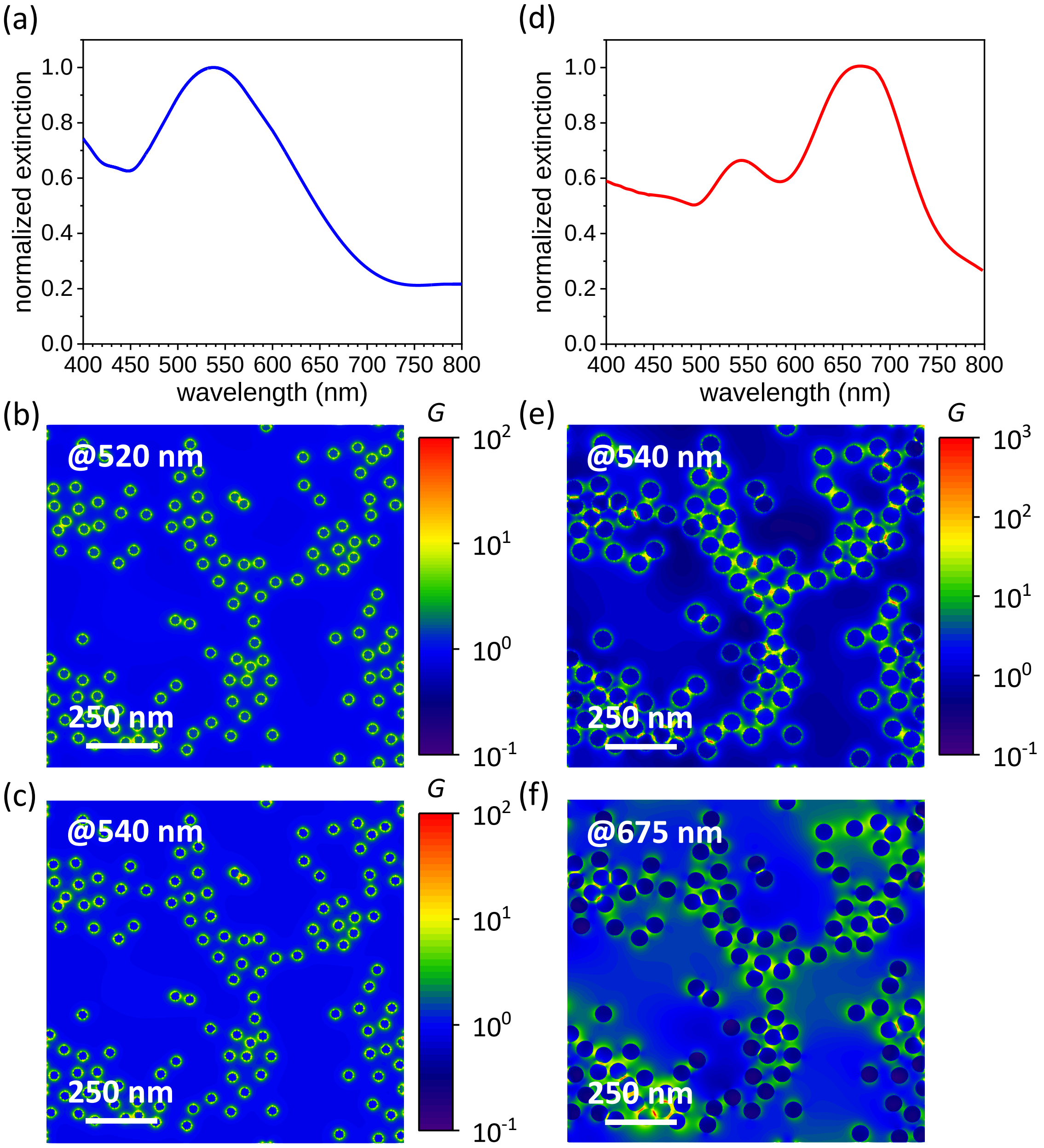

Figure S3. Simulated extinction spectrum of the substrate in the case of homogeneously sized AuNPs of (a) 30 nm diameter and (d) 60 nm diameter. Simulated electric field intensity of the substrate worked out at wavelengths (b) 520 nm and (c) 540 nm in the case of 30 nm diameter AuNPs, and (e) 540 nm and (f) 675 nm in the case of 60 nm diameter AuNPs.

Section S4. Substrate biofunctionalization.

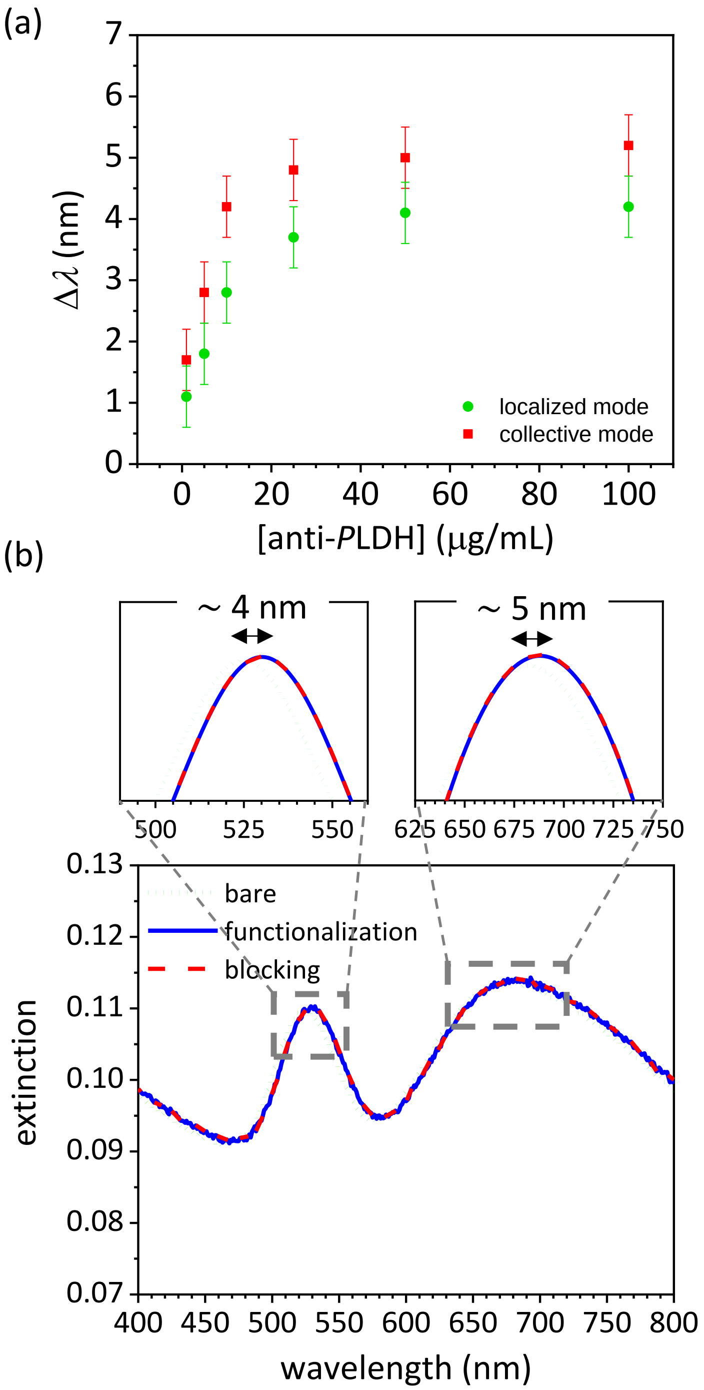

Figure S4. (a) Shift of the plasmon resonance wavelengths ($\Delta\lambda$) as a function of anti-*P*LDH concentration for the localized (green circles) and collective (red squares) modes. Both the resonances red-shift as the anti-*P*LDH concentration increased up to 50 μg/mL that corresponds to a maximum redshift of approximately 4-5 nm. (b) Experimental extinction spectrum of the substrate before (dotted green line) and after functionalization (solid blue line) and blocking (dashed red line). The insets show the Gaussian fits used for evaluating the plasmon resonance wavelength.

Section S5. Fluorescence picture acquisition, processing and analysis.

Fluorescence images were acquired by Zeiss Axio Observer Z1 inverted phase contrast fluorescence microscope equipped by Zeiss Colibri.2 LED light source (modules 470 and 625 nm), Zeiss Plan-Apochromat 10x/0.45 Ph1 M27 (FWD = 2.1 mm) objective, 38 HE filter (excitation 450-490 nm/emission 500-550nm) and cube 50 Cy5 filter (excitation 625-655 nm/emission 665-715 nm), and pco.edge 5.5 sCMOS photodetector (scaling 0.650 µm × 0.650 µm per pixel, image size 2560 × 2160 pixels, scaled image size 1.66 mm × 1.40 mm, 16 bit dynamic range). The camera exposure time was set to 2 s for recording every image.

Raw fluorescence images were processed by ImageJ to measure the whole intensity arising from the bright spots. Firstly, RGB images were split into two channels containing the green and red components so to separately analyse the contribution of the two fluorophores used in this work. Since the raw images contained a smooth continuous background (Figure S5a), the rolling ball algorithm was used to flatten any spatial variations of the background by evaluating the average value over a ball around each pixel and then by locally subtracting such a value from the image (Figures S5b and S5c). Note that the fluorescence intensity was scaled by an arbitrary factor to ease the readability of the outcomes.

Figure S5d shows an example of processed image at higher magnification in which green and red channels are merged and the bright spots strikingly emerge from the background, whereas Figures S5e and S5f depict the corresponding intensity histograms of both the channels. Thus, the whole intensity of 5-FAM and Cy5 fluorophores was measured as a function of the spiked *Pf*LDH concentration by summing the signal contribution of every spot.

Aiming at carrying out a reliable and robust analysis, one different substrate was used for each analyte concentration (rather than exploit additive concentrations on the same substrate), and ten fluorescence images were randomly acquired over each sample thereby obtaining an average value of the whole fluorescence intensity ± standard deviation.

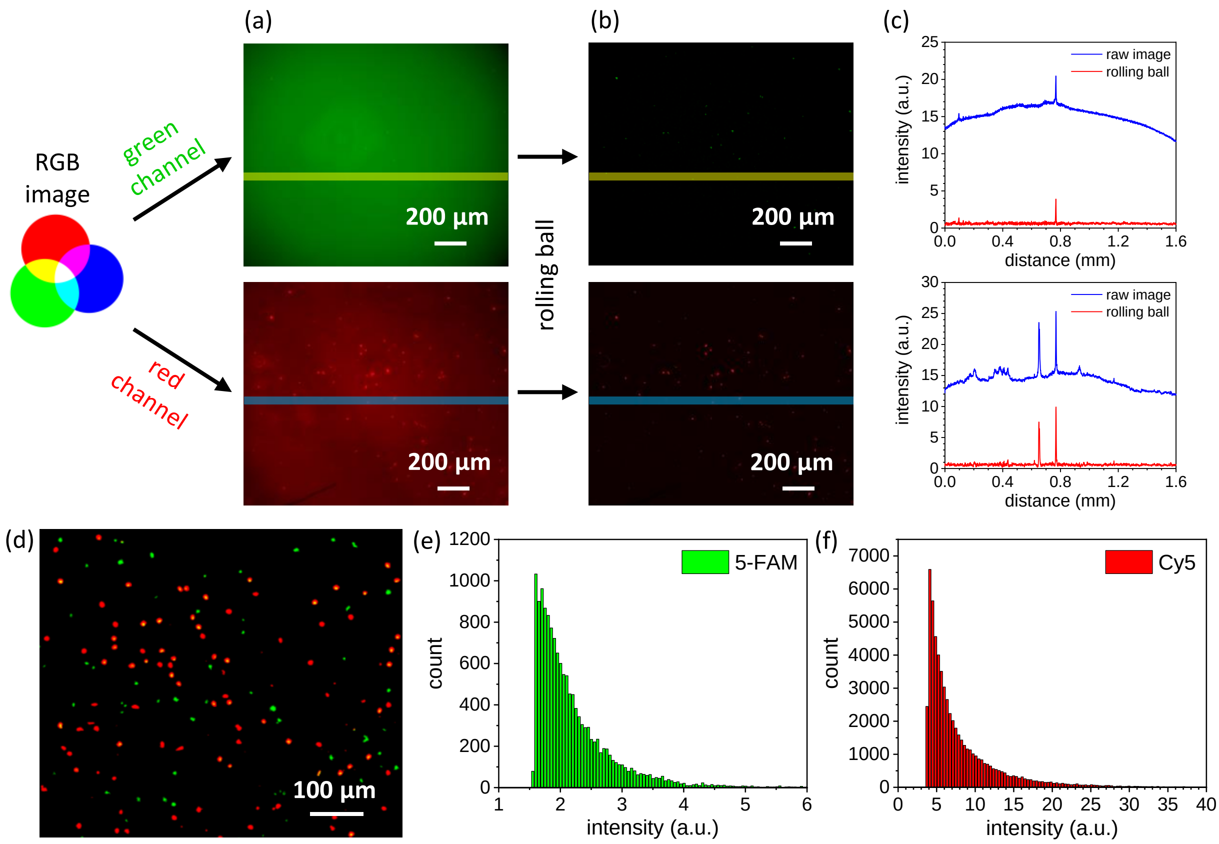

Figure S5. (a) Example of raw fluorescence image split in green channel (top panel) and red channel (bottom panel). The significantly high non-flat background was removed by the rolling ball algorithm. (b) Resulting images exhibit a flat background, from which the fluorescence spots are well distinguishable. (c) Intensity profile evaluated along the yellow and blue lines highlighted in panels (a) and (b), respectively. (d) Example of processed fluorescence image in which the green and red channels are merged again. Corresponding intensity histograms obtained for (e) green channel and (f) red channel.

Section S6. Analysis of the bright spots.

In order to demonstrate the assumption that the fluorescence of each bright spot corresponds to the light emitted by one single fluorophore, we firstly considered the lowest *Pf*LDH concentrations (1 fM) we investigated (Figure 3b in the main text). The corresponding number of analytes in 1 mL volume (i.e., the volume we used throughout the work) is 6×10^5. Thus, in principle we can have 6×10^5 *Pf*LDH proteins over the whole substrate (10×8 mm^2) and, hence, 2×10^4 analytes can be virtually housed in the microscope field of view (FOM) (1.66×1.40 mm^2). In ideal conditions, 2×10^4 fluorophores bind the analytes in the FOM. The photodetector contains 5.5×10^6 pixels (see Section S5).

We can now evaluate the probability that two fluorophores lie on an area of the substrate whose fluorescence would be collected by one pixel (0.650×0.650 μm^2, see Section S5). Since the number of fluorophores (2×10^4) is much smaller than the number of pixels (5.5×10^6), we can adopt the Poisson statistics to retrieve the probability $P(k)$ that k fluorophores are placed in one pixel[4]

$$P(k) = \mathrm{e}^{-\frac{m}{n}} \frac{\left(\frac{m}{n}\right)^k}{k!} \tag{1}$$

where m is the number of fluorophores and n is the number of pixels. It results $P(2)$ = 0.0005%. Therefore, we can safely assert that bright spots appearing in fluorescence images at very low *Pf*LDH concentration (femtomolar level) arise from individual fluorophores.

Moving to the next *Pf*LDH concentrations we explored 1 pM, 1 nM, and 1 μM, the number of fluorophores housed in the FOM in ideal conditions is 2×10^7, 2×10^{10}, and 2×10^{13}, respectively. Since $m > n$, we evaluated the probabilities $P(0)$ that one pixel is vacant. Since $P(0) = \mathrm{e}^{-m/n}$, it results 43% at 1 pM and negligible values at 1 nM and 1 μM. However, considering l dark pixels, the probabilities are much lower $P^{(l)}(0) = \mathrm{e}^{-ml/n}$. For instance, $P^{(10)}(0)$ = 0.02% at 1 pM. Nevertheless, in real measurements a much larger number of dark pixels were present as compared to the number of bright spots even at the largest *Pf*LDH concentration (Figure 3b in the main text). This result can be reasonably achieved only if $m \ll n$.

Such a discrepancy can be justified considering a low system efficiency that depends on (i) the binding affinities between Abs-Au, Abs-*Pf*LDH and Apts*-*Pf*LDH and (ii) the fluidic efficiency in each step. Thus, only a small amount of the available fluorophores would be housed on the substrate and only a tiny fraction of them would experience a significant FE. Therefore, we can assume that the condition $m < n$ is hold even at large concentrations (up

to micromolar level) and, hence, the probability $P(l)$ that $l \geq 2$ fluorophores lie on one pixel is low.

The previous argumentation is coherent with the linearity exhibited by the correlation curves fluorescence *versus* spot area (Figure S6). The slope of the linear regressions is independent from the *Pf*LDH concentration revealing that the mean fluorescence intensity per pixel at low analyte concentrations (femtomolar level) is hold even at high concentrations (micromolar level). In addition, the number of bright spots whose intensity/area ratio lies outside the linear regression is relatively low even if it enlarges as the analyte concentration increases. In particular, these spots arose from nearby fluorophores whose light was collected by the same pixels of the photodetector so to increase the spot brightness while holding the spot size. In conclusion, we can safely assert that most of the observed bright spots arise from individual fluorophores.

Finally, we can retrieve the ratio between the measured FE (of approximately 28) by considering the ratio between the slopes of the corresponding correlation curves (fluorescence vs spot area)

$$\frac{\langle FE_{\mathrm{Cy5}}^{\mathrm{obs}} \rangle}{\langle FE_{\mathrm{5FAM}}^{\mathrm{obs}} \rangle} \sim \frac{m_{\mathrm{Cy5}}}{QY_{\mathrm{Cy5}}^{0}} \frac{QY_{\mathrm{5FAM}}^{0}}{m_{\mathrm{5FAM}}} \tag{2}$$

where $QY_{\mathrm{5FAM}}^{0} = 0.83$[5] and $QY_{\mathrm{Cy5}}^{0} = 0.27$.[6] Thus, we obtain a value of ~20 by Equation (2) that is consistent with that evaluated by considering the measured FE.

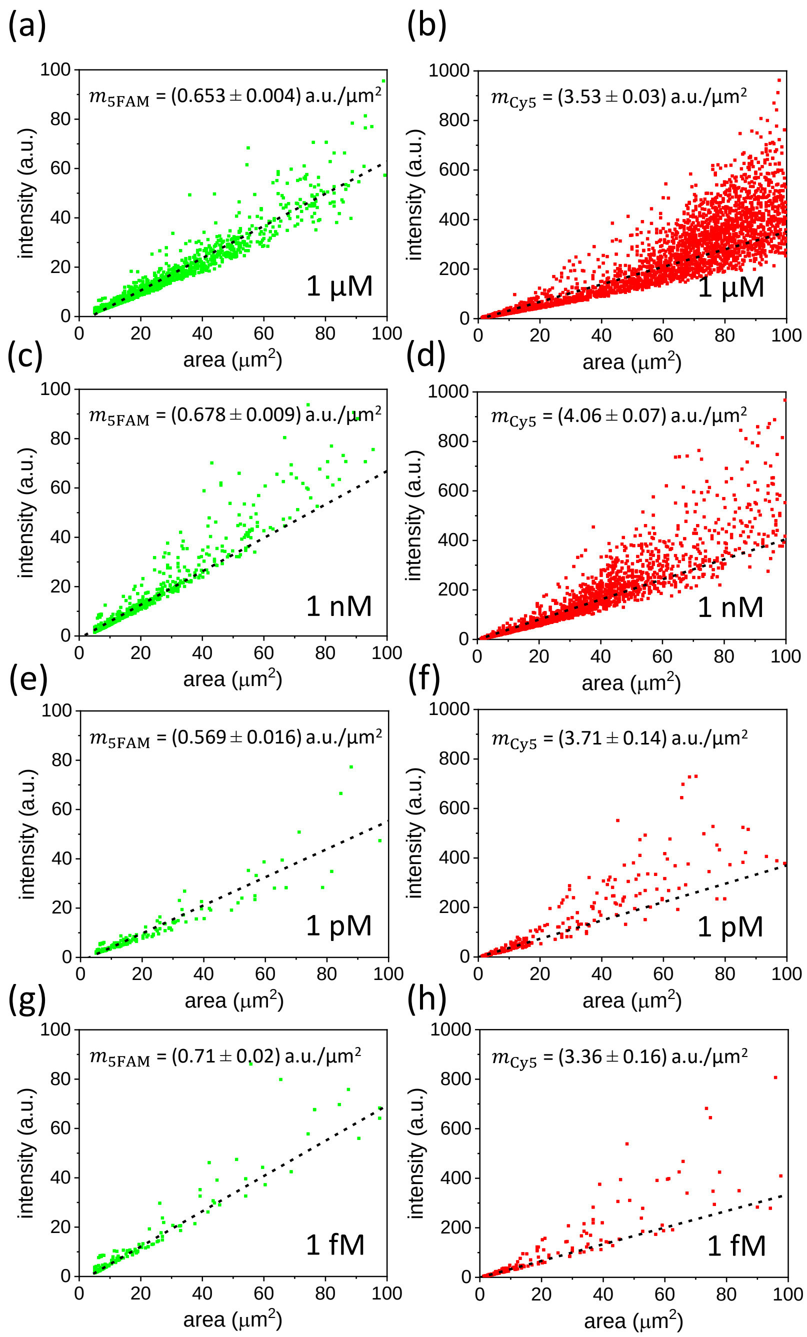

Figure S6. Correlation intensity vs spot area explored by (a,c,e,g) 5-FAM dye and (b,d,f,h) Cy5 dye at *Pf*LDH concentrations of (a,b) 1 µM, (c,d) 1 nM, (e,f) 1 pM and (g,h) 1 fM spiked in human whole blood (Pearson's *r* = 0.95).

Section S7. Off-resonance simulations.

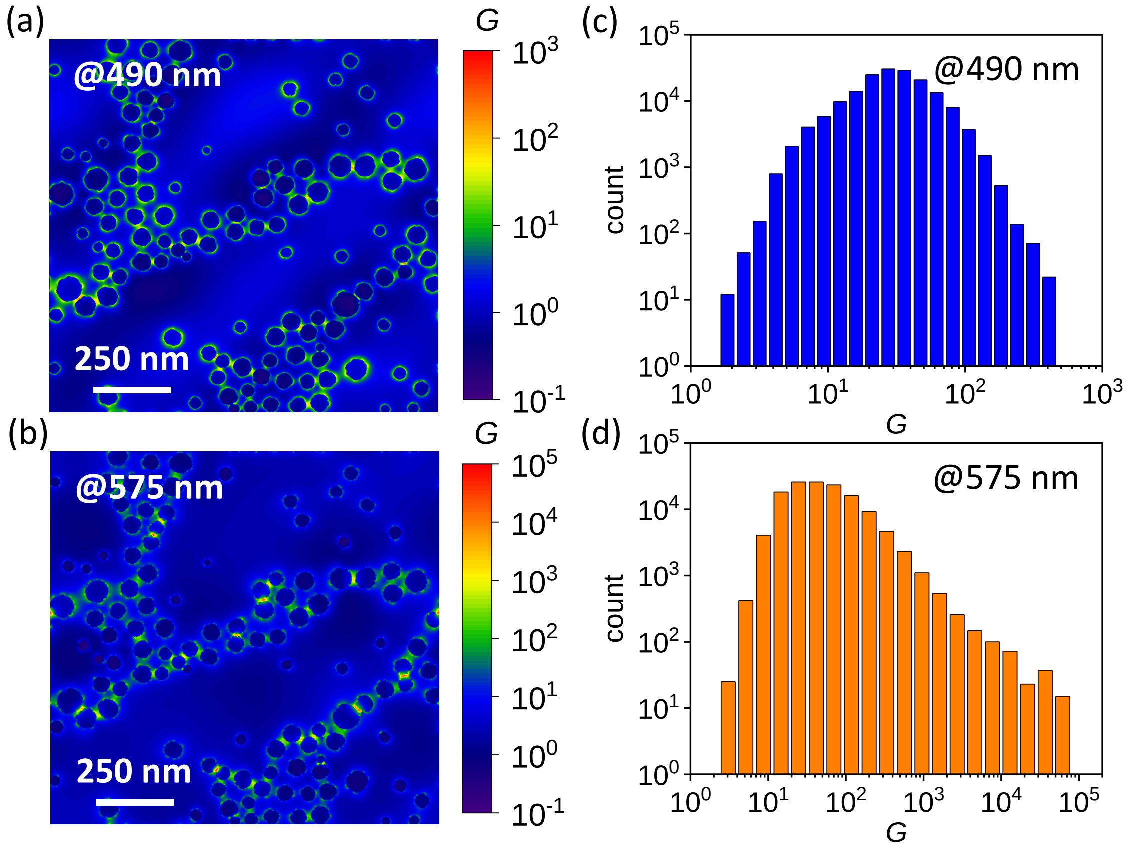

Figure S7. Simulated electric field intensity of the substrate worked out at wavelength (a) 490 nm and (b) 575 Histograms of the G value distribution evaluated in annulus-shaped regions around nanoparticles (5 nm thickness, 5 nm offset from particle surface) at wavelength (c) 490 nm and (d) 575 nm.

Section S8. Analysis of the electric field.

The electric field E_n around the n-th nanoparticle was evaluated in the x-y plane in annuluses of 5 nm thickness and 5 nm offset from the nanoparticle surface by Equation (3)

$$E_n(r,\varphi) = E(x_n + r\cos\varphi, y_n + r\sin\varphi) \quad (3)$$

where r is the distance from the nanoparticle centroid (1 nm spatial resolution), φ is the azimuthal angle (1° angular resolution), and (x_n, y_n) are the centroid coordinates. Note that r values range from $D_n/2$ + 5 nm to $D_n/2$ + 10 nm, in which D_n is the diameter of the n-th nanoparticle measured by SEM micrographs (see Section S1). We implemented Equation (3) in a Python code that excluded multiple counting in overlapping annuluses. Figure S8 shows two representative magnifications of the electric field intensity worked out with perfectly spherical gold nanoparticles and rough gold nanoparticles, in which the shaded red annuluses highlight the only regions where the G values were evaluated.

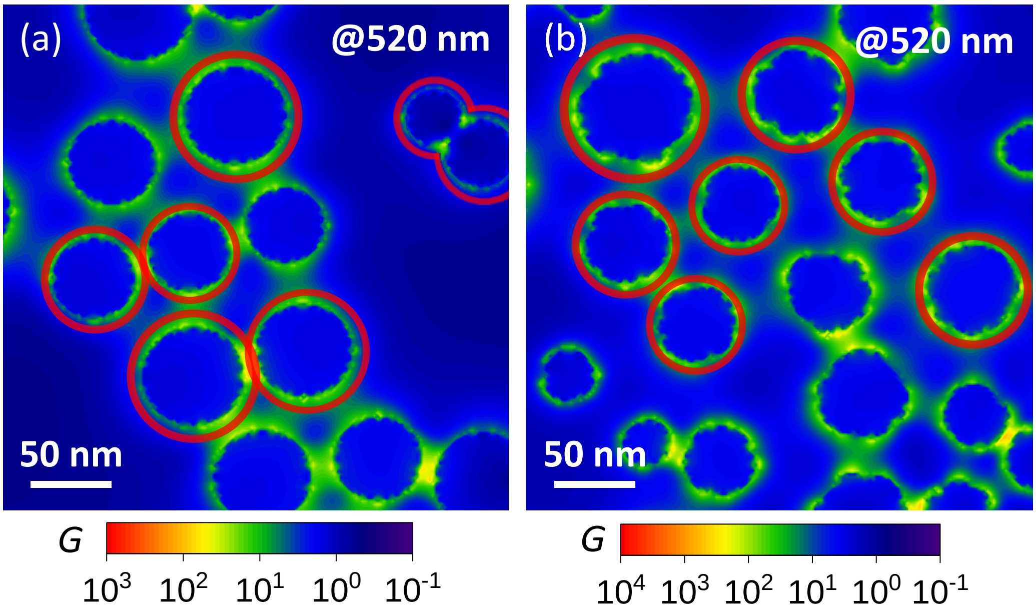

Figure S8. Electric field intensity distributions of (a) perfectly spherical gold nanoparticles and (b) rough gold nanoparticles worked out at 520 nm wavelength. The shaded red annuluses (5 nm thickness, 5 nm offset from the nanoparticle surface) highlight the regions where the G values were evaluated. Note that in the case of rough nanoparticles, the annulus offset is determined by considering an ideal spherical surface embedding the nanoparticle roughness.

Section S9. Electromagnetic response of the substrate with perfectly spherical nanoparticles.

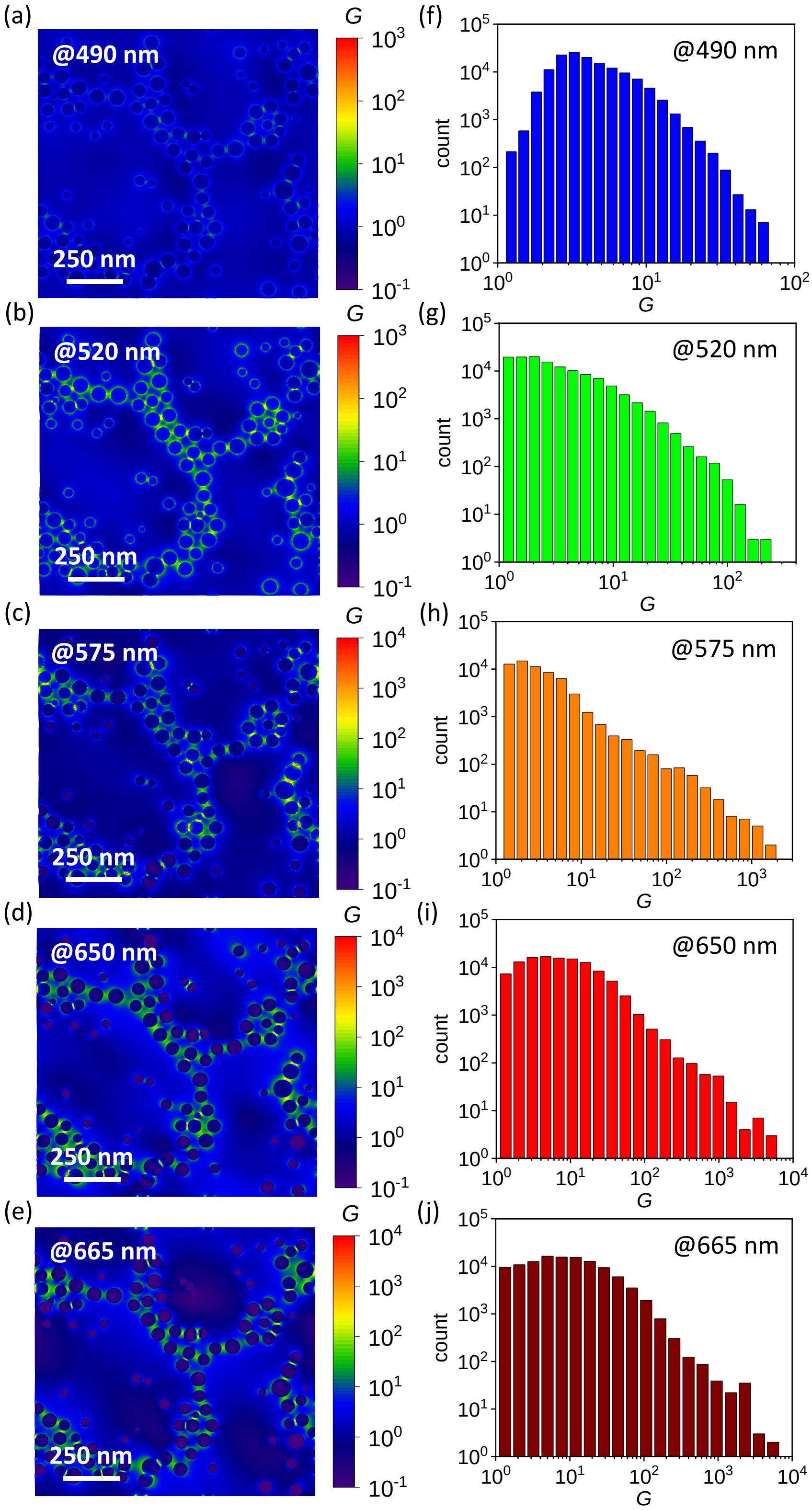

Figure S9. Simulated electric field intensity of the substrate in the case of perfectly spherical AuNPs worked out at wavelength (a) 490 nm, (b) 520 nm, (c) 575 nm, (d) 650 nm, and (e) 665 nm. Histograms of the *G* value distribution evaluated in annulus-shaped regions around nanoparticles (5 nm thickness, 5 nm offset from particle surface) at wavelength (f) 490 nm, (g) 520 nm, (h) 575 nm, (i) 650 nm, and (j) 665 nm.

Section S10. Electromagnetic response of non-spherical nanoparticles.

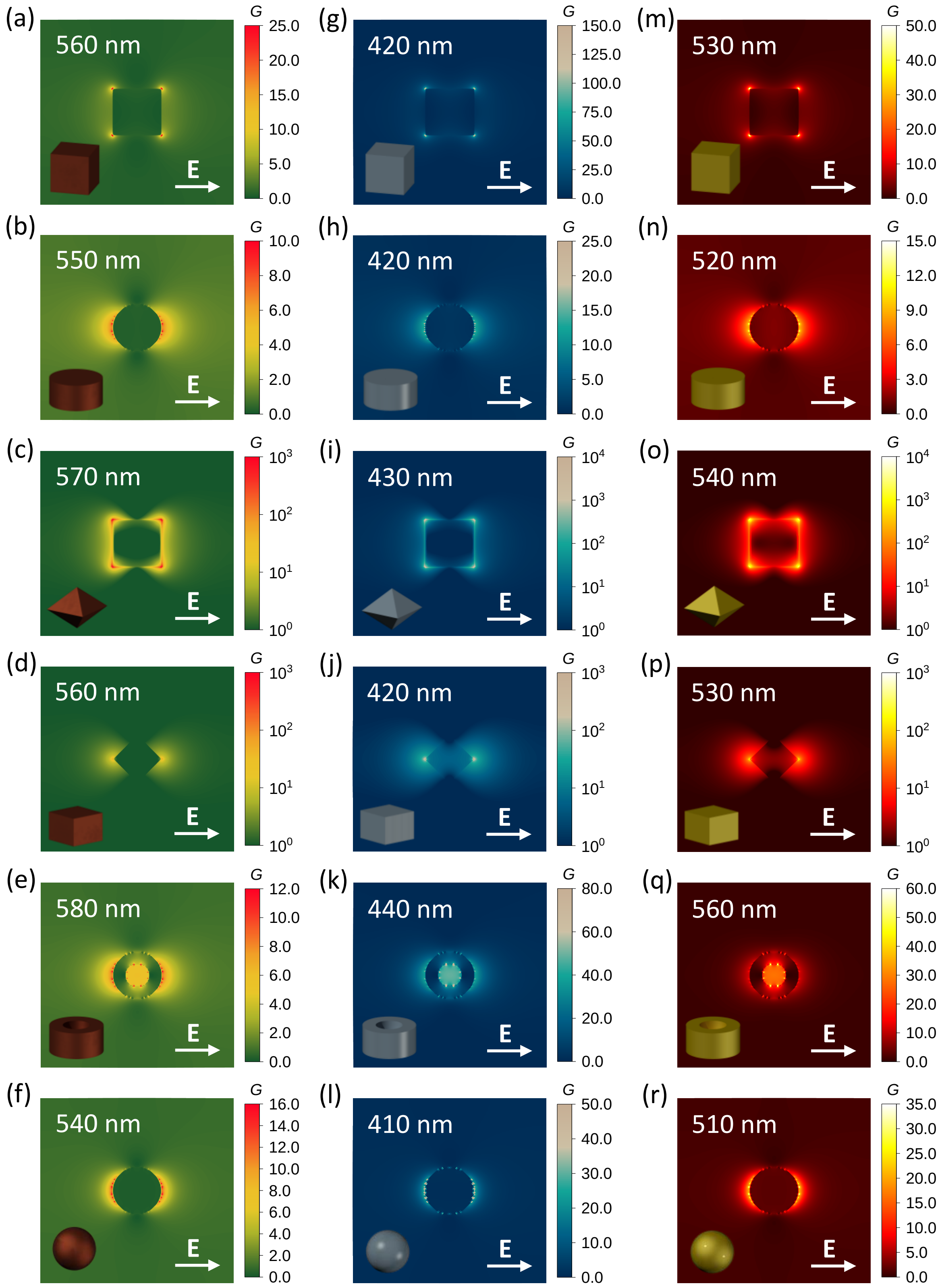

Figure S10.A. Simulated electric field intensity of a copper (left panels), silver (middle panels), gold (right panels) (a,g,m) cube, (b,h,n) disk, (c,i,o) octahedron, (d,j,p) rhombus, (e,k,q) ring, and (f,l,r) sphere worked out at the corresponding resonance wavelength.

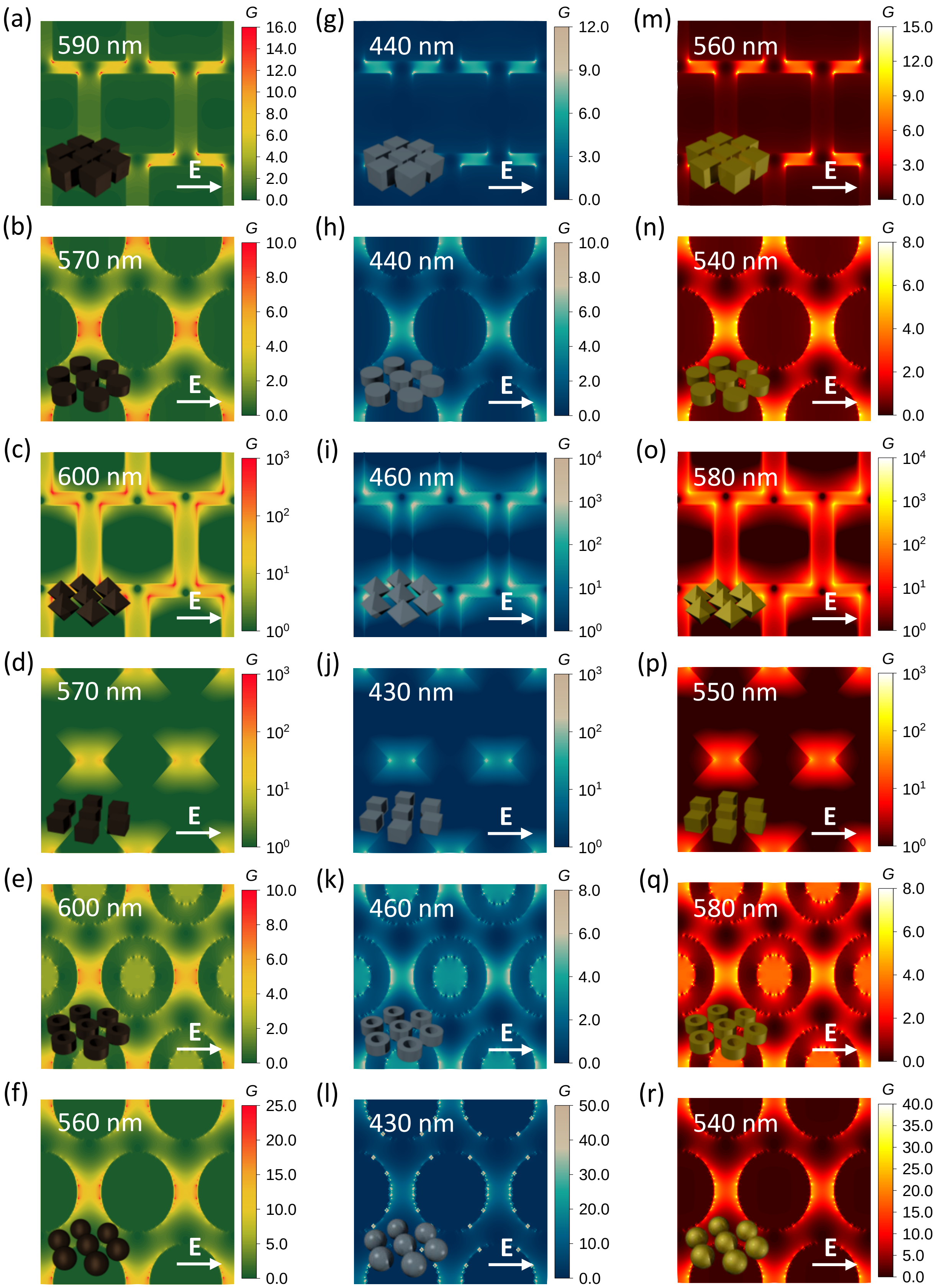

Figure S10.B. Simulated electric field intensity of a hexagonal lattice of copper (left panels), silver (middle panels), gold (right panels) (a,g,m) cubes, (b,h,n) disks, (c,i,o) octahedra, (d,j,p) rhombi, (e,k,q) rings, and (f,l,r) spheres worked out at the corresponding resonance wavelength.

Section S11. Fluorescence intensity in non-PEF conditions.

To measure the intensity $\langle I_0 \rangle$, a volume of 100 μL containing 250 fmol of Apts* (approximately 1.5 × 10^{11} fluorophores) was drop-casted onto a microscope slide. After drying, the 5-FAM (Cy5) fluorophores resulted to be confined within a circular region of 12 mm (13 mm) diameter surrounded by an annulus (i.e., coffee ring) of 0.15 mm (0.10 mm) thickness whose intensity was 10-fold (4-fold) higher than that measured in the inner region (Figure S11).

The whole fluorescence intensity F_0 of the dried drop was measured by sampling the drop area in 100 regions of size 350 × 350 μm^2 in the inner region and 70 × 70 μm^2 on the annulus. Then, the resulting averages were scaled to the whole inner region (~113 mm^2 for 5-FAM dye and ~133 mm^2 for Cy5 dye) and coffee ring area (~5.7 mm^2 for 5-FAM dye and ~4.1 mm^2 for Cy5 dye). Thus, the mean intensity $\langle I_0 \rangle$ provided by individual fluorophores in free-space condition is

$$\langle I_0 \rangle = \frac{F_0}{N_0} \tag{4}$$

where N_0 the number of fluorophores contained in the drop.

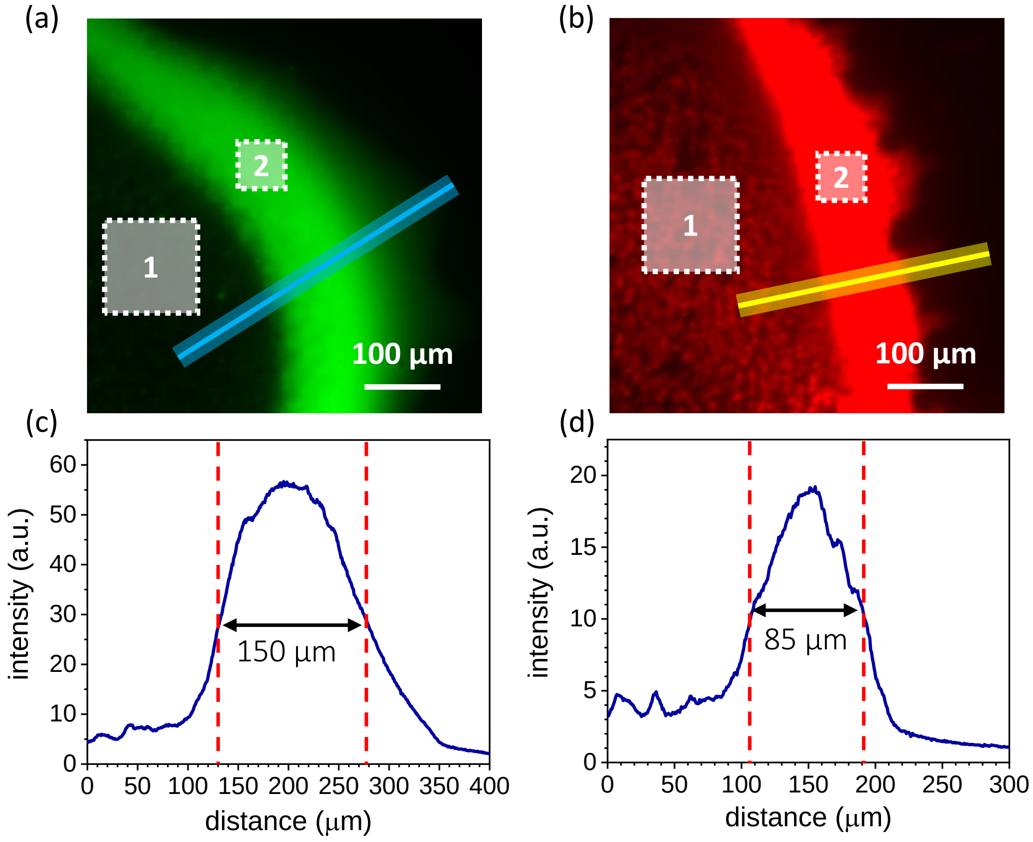

Figure S11. Fluorescence image of (a) 5-FAM-Apts* and (b) Cy5-Apts* dried drops. The drop was sampled in 100 regions of size 350 × 350 μm^2 in the inner area (white squares 1, not in scale) and 70 × 70 μm^2 on the annulus (white squares 2). (c,d) Ring thickness measured as FWHM of the intensity profile evaluated along the light blue and yellow lines highlighted in panels (a) and (b), respectively. Adapted under the terms of the CC-BY license.[7,8] Copyright 2020 and 2021, The Authors, published by Springer Nature.

Section S12. Chemicals and materials.

Gold(III) chloride trihydrate ($HAuCl_4 \cdot 3H_20$), silver nitrate ($AgNO_3$), ascorbic acid and toluene (99.8%) were purchased from Sigma-Aldrich; ethanol (≥ 99.5%), 2-propanol (≥ 99.5%) and acetone (≥ 99.0%) were purchased from Merck Millipore; hexadecyltrimethylammonium bromide (CTAB) (≥ 99.0%) was purchased from Fluka; bovine serum albumin (BSA) (fraction V IgG free, fatty acid poor) was purchased from Gibco. Diblock copolymers (P3807-S2VP) were purchased from Polymer Source Inc. (Dorval, Canada) and were made by polystyrene(*x*)-b-2-poly-vinylpyridine(*y*) (PS(*x*)-b-P2VP(*y*)), in which x = 325000 g/mol and y = 92000 g/mol are the molecular weight of polystyrene (PS) and poly(2-vinylpyridine) (P2VP), respectively. Ultrapure deionized water used for all aqueous solutions was dispensed by Milli-Q® system (18.2 MΩ cm resistivity). 10 mM phosphate-buffered saline (PBS) (NaCl 10 mM, NaH_2PO_4 10 mM, Na_2HPO_4 10 mM, $MgCl_2$ 1 mM, pH 7.1) and 25 mM Tris-HCl buffer (NaCl 100 mM, imidazole 20 mM, Tris 25 mM, HCl 25 mM, pH 7.5) were prepared by dissolving the reagents (purchased from Sigma-Aldrich) in ultrapure water. Pan malaria antibody (monoclonal anti-*P*LDH antibody clone 19g7) was produced by Vista Laboratory Services (Langley, USA). The recombinant *Pf*LDH and *Pv*LDH were obtained from bacterial expression as previously described.[9] The malaria 2008s aptamer labelled with 5-FAM (analogously, Cy5) tag (5'-5-FAM(Cy5)-CTG GGC GGT AGA ACC ATA GTG ACC CAG CCG TCT AC-3') was synthesized by Friz Biochem GmbH (Neuried, Germany). Millex® syringe filters (pore size 0.20 µm) with hydrophilic polytetrafluoroethylene membrane were purchased from Merck Millipore; Superslip™ cover slips (borosilicate glass, thickness 0.13-0.17 mm) were purchased from Thermo Fisher Scientific.

Section S13. Photochemical immobilization technique.

The PIT consists of UV irradiation of Abs that produces the selective photoreduction of the disulfide bridge in specific cysteine-cysteine/tryptophan (Cys-Cys/Trp) triads,[10] which are a typical structural feature of the immunoglobulin G (IgG).[11] The UV excitation of the Trp residue leads to the generation of solvated electrons that are captured by the nearby disulphide bridge resulting in its destabilization and subsequent breakage of the Cys-Cys bond. The breakage of such Cys-Cys bonds in both Ab Fab fragments produces four free thiol groups (Figure S12a), two of which are able to interact with the proximal gold surface giving rise to a covalent Ab tether. Functionalization by PIT assures both close-packing and control over the orientation of the immobilized Abs, with one of their binding sites exposed to the surrounding environment (Figure S12b).

The UV source (Trylight, Promete S.r.l.) consisted of two U-shaped low-pressure mercury lamps (6 W at 254 nm) in which a standard 10 mm quartz cuvette could be easily housed (Figure S12c). By considering the wrapping geometry of the lamps and the proximity of the cuvette, the irradiation intensity used for the thiol group production was approximately 0.3 W/cm^2. Such an intensity is low enough to avoid any significant photolysis of the disulfide bridge that poorly absorbs at 254 nm.[12]

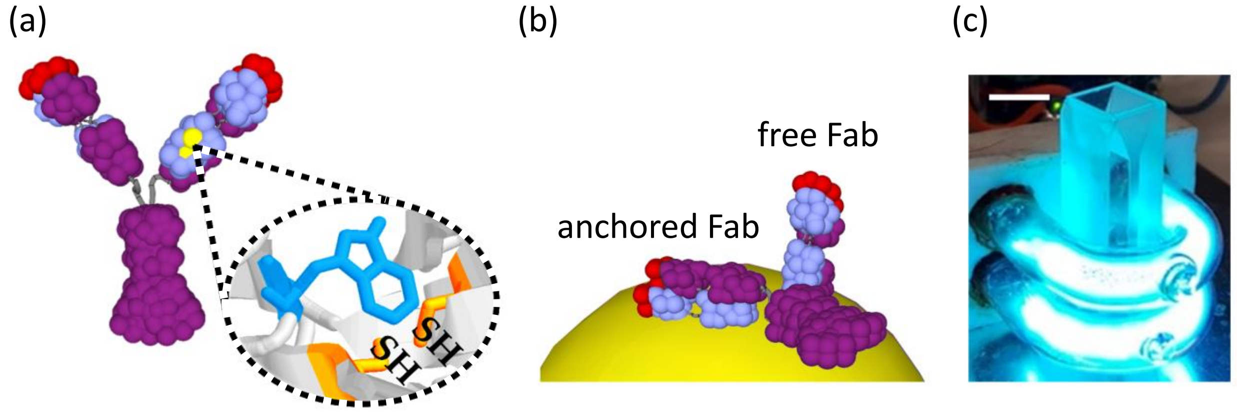

Figure S12. (a) UV irradiation of Abs leads to the selective production of four thiol groups (two of them are not visible in the figure). (b) The opposite position of the thiols with respect to the plane containing the Fabs allows the Abs to be immobilized with one of their binding sites exposed to the surrounding environment. (c) Low pressure mercury U-shaped UV lamps used to carry out the thiol activation in antibody Fabs. The length of the scale bar in the top-left corner is 1 cm. Adapted under the terms of the CC-BY license.[8] Copyright 2021, The Authors, published by Springer Nature.

Section S14. Microfluidic system.

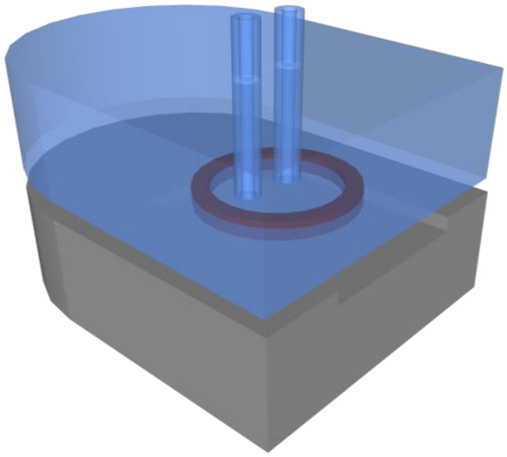

Figure S13. Sketch of the fluidic cell used to promote an effective interaction of the irradiated Abs contained into the aqueous solution with the nanostructured substrate. Adapted under the terms of the CC-BY license.[8] Copyright 2021, The Authors, published by Springer Nature.

Section S15. Preparation of buffer solutions.

Table S2. Tris buffer solution was prepared by dissolving the reagents into 500 mL of ultrapure water under vigorous stirring at room temperature. The buffer pH was adjusted to 7.5 by adding 750 µL of HCl 37%.

Chemical	State	Added amount	Final concentration
NaCl	powder	2.922 g	100 mM
Imidazole	powder	0.681 g	20 mM
Tris base	powder	1.515 g	25 mM
HCl 37%	liquid	397 µL	-

Table S3. PBS solution was prepared by dissolving the reagents into 100 mL of ultrapure water under vigorous stirring at room temperature. The buffer pH was adjusted to 7.1 by adding 250 µL of NaOH (4 M).

Chemical	State	Added amount	Final concentration
NaCl	powder	585 mg	100 mM
NaH_2PO_4	powder	140 mg	10 mM
Na_2HPO_4	powder	140 mg	10 mM
$MgCl_2$	powder	9.5 mg	1 mM

References

(1) Haynes, W. M. *CRC Handbook of Chemistry and Physics*; Haynes, W. M., Lide, D. R., Bruno, T. J., Eds.; CRC Press, 2016. https://doi.org/10.1201/9781315380476.

(2) Palik, E. D. *Handbook of Optical Constants of Solids*; Elsevier, 1985. https://doi.org/10.1016/C2009-0-20920-2.

(3) Bérenger, J. P. Perfectly Matched Layer (PML) for Computational Electromagnetics. *Synth. Lect. Comput. Electromagn.* **2007**, *8* (1), 1–120. https://doi.org/10.2200/S00030ED1V01Y200605CEM008.

(4) Raab, M.; Steger, A. “Balls into Bins” — a Simple and Tight Analysis. In *Lecture Notes in Computer Science (including subseries Lecture Notes in Artificial Intelligence and Lecture Notes in Bioinformatics)*; Luby, M., Rolim, J. D. P., Serna, M., Eds.; Springer Berlin Heidelberg: Berlin, Heidelberg, 1998; Vol. 1518, pp 159–170. https://doi.org/10.1007/3-540-49543-6_13.

(5) Povedailo, V. A.; Lysenko, I. L.; Tikhomirov, S. A.; Yakovlev, D. L.; Tsybulsky, D. A.; Kruhlik, A. S.; Fan, F.; Martynenko-Makaev, Y. V.; Sharko, O. L.; Duong, P. V.; et al. Fluorescent Properties of Carboxyfluorescein Bifluorophores. *J. Fluoresc.* **2020**, *30* (3), 629–635. https://doi.org/10.1007/s10895-020-02535-w.

(6) Mujumdar, R. B.; Ernst, L. A.; Mujumdar, S. R.; Lewis, C. J.; Waggoner, A. S. Cyanine Dye Labeling Reagents: Sulfoindocyanine Succinimidyl Esters. *Bioconjug. Chem.* **1993**, *4* (2), 105–111. https://doi.org/10.1021/bc00020a001.

(7) Minopoli, A.; Della Ventura, B.; Lenyk, B.; Gentile, F.; Tanner, J. A.; Offenhäusser, A.; Mayer, D.; Velotta, R. Ultrasensitive Antibody-Aptamer Plasmonic Biosensor for Malaria Biomarker Detection in Whole Blood. *Nat. Commun.* **2020**, *11* (1), 6134. https://doi.org/10.1038/s41467-020-19755-0.

(8) Minopoli, A.; Della Ventura, B.; Campanile, R.; Tanner, J. A.; Offenhäusser, A.; Mayer, D.; Velotta, R. Randomly Positioned Gold Nanoparticles as Fluorescence Enhancers in Apta-Immunosensor for Malaria Test. *Microchim. Acta* **2021**, *188* (3), 88. https://doi.org/10.1007/s00604-021-04746-9.

(9) Cheung, Y.-W.; Dirkzwager, R. M.; Wong, W.-C.; Cardoso, J.; D’Arc Neves Costa, J.; Tanner, J. A. Aptamer-Mediated Plasmodium-Specific Diagnosis of Malaria. *Biochimie* **2018**, *145*, 131–136. https://doi.org/10.1016/j.biochi.2017.10.017.

(10) Neves-Petersen, M. T.; Gryczynski, Z.; Lakowicz, J.; Fojan, P.; Pedersen, S.; Petersen, E.; Bjørn Petersen, S. High Probability of Disrupting a Disulphide Bridge Mediated by an Endogenous Excited Tryptophan Residue. *Protein Sci.* **2002**, *11* (3), 588–600.

https://doi.org/10.1110/ps.06002.

(11) Ioerger, T. R.; Du, C.; Linthicum, D. S. Conservation of Cys–Cys Trp Structural Triads and Their Geometry in the Protein Domains of Immunoglobulin Superfamily Members. *Mol. Immunol.* **1999**, *36* (6), 373–386. https://doi.org/10.1016/S0161-5890(99)00032-2.

(12) Neves-Petersen, M. T.; Klitgaard, S.; Pascher, T.; Skovsen, E.; Polivka, T.; Yartsev, A.; Sundström, V.; Petersen, S. B. Flash Photolysis of Cutinase: Identification and Decay Kinetics of Transient Intermediates Formed upon UV Excitation of Aromatic Residues. *Biophys. J.* **2009**, *97* (1), 211–226. https://doi.org/10.1016/j.bpj.2009.01.065.
